# Microbial intervention on germ-free zebrafish (*Danio rerio*) developmental delay and gut-brain axis hormones disturbance from innate to adaptive immune stages

**DOI:** 10.3389/fimmu.2026.1930616

**Published:** 2026-10-19

**Authors:** Lan-Chen Zhang, Hao-Yu Yang, Jin-Qiu Jia, Xiao-Bing Li, Jia-Xin He, Ying Tang, Yi-Wei Tang, Pan-Pan Jia

**Affiliations:** 1 College of Public Health, Chongqing Medical University, Chongqing, China; 2 Science and Technology Industry Development Center, Chongqing Medical and Pharmaceutical College, Chongqing, China

**Keywords:** developmental delay, diverse life stages, germ-free zebrafish, gut-brain axis hormones, microbial intervention

## Abstract

**Introduction:**

The gut microbiota is associated with host development and endocrine signaling, but its role in early zebrafish development remains incompletely understood. Germ-free (GF) fish models are powerful tools to reveal the functions of intestinal microbiota and special strains.

**Methods:**

This study generated and identified different models of conventionally raised (CR) fish, fish recolonized with a conventional microbial community (ex-GF), and GF fish infected with *Aeromo*nas sp. (GF-A) or *Vibrio* sp. (GF-V).

**Results:**

The delayed development indexes assessed at 7 and 14 days-post fertilization (dpf) showed an obviously weaker behavior ability of GF fish without microbiota. Moreover, the histopathological characteristics indicated defects in intestinal morphology, fewer goblet cells, and ultrastructural abnor-malities using H&E and AB-PAS stains and ultra-structure by TEM analyses. The transcriptomic profile and gut microbiota also revealed differentially expressed genes (DEGs) and the dominant genera of GF or ex-GF larvae and juveniles, with the most impacted pathways being immune and cell regulation. Related hormones, short-chain fatty acid concentrations, and key genes were detected and disruption was exhibited by the absence or recolonization of gut microbiota or infection of special bacterial strains. Transcriptomic, microbial, and endocrine alterations differed among groups. Selected phenotypes were partially improved with stage-dependent restructuring but did not completely resemble CR states across all endpoints. The correlation analysis indicated the close relationship between developmental delay and microbiota depletion, behavior, hormone contents, and key genes in regulating pathways.

**Conclusion:**

These findings demonstrate that microbial intervention contributes to early development of zebrafish, which may correlate with the gut-brain axis hormones. Thus, it contributes to the understanding of the roles of microbiota for diverse life stages and contributes to the development of microbial therapies in future.

## 1 Introduction

Regarded as a “hidden organ” and a “second genome”, the gut microbiota regulates host growth and development, metabolism, immune system maturation, and intestinal homeostasis via the gut–brain axis (1, 2). Interaction between the gut-brain axis can happen through neural, endocrine, or immune regulatory pathways and contribute to host metabolism, signal transduction, immunity, and the functioning of important organs such as the intestine, liver, and brain (3). In endocrine regulatory pathways, the gut-brain axis releases various hormones and polypeptide substances to jointly impact host growth and development, hunger, energy maintenance, and circadian rhythms (4, 5). These key hormones include the corticotropin-releasing hormone (CRH), thyrotropin-releasing hormone (TRH), thyroid stimulating hormone (TSH), serotonin (known as 5-hydroxytryptamine, 5-HT), glucagon-like peptide (GLP-1), cholecystokinin (CCK), peptide tyrosine-tyrosine (PYY), ghrelin, leptin (LEP), growth hormone-insulin-like growth factor (GH/IGF) axis, and hypothalamic-pituitary-thyroid (HPT) axis related growth hormone (GH) and thyroid hormones (THs) (6–10). However, the interaction between microbial intervention and host hormones during early vertebrate development still remains incompletely revealed.

Zebrafish (*Danio rerio*) have been widely used as credible models in environmental toxicology, disease, drug screening, and biomedical research (11, 12). Germ-free (GF) zebrafish can provide a tractable system for examining associations between microbial colonization and host growth and development, such as digestion, metabolism, and hormone secretion (13–15). During the early periods of an animal’s life, dysbiosis of the microbiota can affect the gut-brain axis, but restoration can significantly improve the transcriptomic profile and anxiety behavior (16, 17). Zebrafish larvae at early life (7 dpf) with innate immunity develop a functional adaptive immune system at the juvenile stage (14–21 dpf), thus GF models have been applied to study microbial regulation from the innate to adaptive immune stages (12, 18, 19). Thus, the high-throughput behavior, transcriptomic profile, and hormones analyses by comparing the GF zebrafish and related models were performed to clearly elucidate the regulation and inner mechanism of diverse microbial intervention strategies.

The gut microbiota interacts with hormone secretion, exhibiting a bidirectional regulatory relationship with host growth and development (20, 21). In the GH/IGF axis, growth hormone (GH) significantly influences fish growth through factors such as the growth hormone receptor (GHR), insulin-like growth factor (IGF), and insulin-like growth factor receptor (IGFR) (22). GH (*gh*) secreted by the pituitary gland can bind to the corresponding receptors (*ghra*, *ghrb*) in the liver and promote the synthesis and secretion of insulin-like factors IGF (*igf1*, *igf2*), which subsequently interacts with the corresponding binding receptors IGFR (*igf1ra*, *igf1rb*) to increase body length, weight, *etc. (*23). Thyroid hormones (THs) secreted by the HPT axis promote organ growth and metamorphic development from embryos to juvenile fish (24–26). Furthermore, the intestinal microbiota also regulate hormones such as 5-HT, GLP-1, CCK, PYY, ghrelin, and leptin which interact with each other *via* the gut-brain axis endocrine system, influencing host behaviors, metabolism, appetite, energy balance, growth and development, reproduction, and immune functions (27–29). Of these hormones, 5-HT is an important neurotransmitter, 90% of which is produced in the digestive tract, and can affect the host’ intestinal motility, secretion, appetite, and nutrient absorption (30). Microbially derived short-chain fatty acids (SCFAs) are important signaling molecules in the gut-brain axis to stimulate the secretion of 5-HT, PYY, and GLP-1, and then to regulate host physiological processes by probiotic effect on improving the intestinal homeostasis (31–33). Therefore, the correlation between these gut-brain axis hormones and the delayed development were explored to reveal the effects of microbial intervention on GF zebrafish.

Early life is an important period for initial microbial colonization or composition and is essential for host growth and development, resistance to pathogens, maturation of the immune system, and establishment of the metabolism (34, 35). Disordered or delayed colonization of early intestinal flora was one factor leading to growth delay in children, while a reasonable flora structure promoted health (36). Probiotics have been shown to improve the host health status, and the library of bacteria isolated from gut microbiota is of great significance for mining of probiotic strains (37, 38). Microbial communities that colonize the intestines of early juvenile fish mainly come from the water environment in which they live (39). Previously, we utilized intestinal bacteria, including genera *Pseudomonas*, *Aeromonas*, and *Vibrio*, with high abundance and diverse metabolic functions to further infect GF zebrafish and found it promoted the host’s immune system and intestinal goblet cells (12, 40, 41). Therefore, these isolated bacteria can be used as experimental mono-association strains to compare specific bacterial infection with conventional community recolonization, which may contribute to insights into the microbial therapeutic or probiotic agents.

This study generated and compared conventionally raised (CR), germ-free (GF), recolonized (ex-GF), and gut bacteria–infected (Aeromonas or Vibrio; GF-A, GF-V) models across the innate and adaptive immune developmental stages. The effects of microbiota on regulatory gut-brain axis hormones and important metabolites were explored to see whether microbial intervention can contribute to the improvement of host growth and homeostasis. Further, a correlation analysis between key indexes of development, behavior, intestinal morphology, microbiota structure, gut-brain axis hormones, metabolites, and transcriptomic changes were conducted, which screened potential “biomarkers” for microbial regulation on host developmental delay and immune defects.

## 2 Methods and materials

### 2.1 Ethics approval and consent to participate

The animal experiments on zebrafish were approved by the Animal Care and Use Committee of Chongqing Medical University (Chongqing, China), and referred to the Administration of Experimental Animals issued by the Ministry of Science and Technology, and standards for experimental animals issued by the State Bureau of Quality and Technical Supervision (Approval ID: GB14922–2001 to GBT14927-2001).

### 2.2 Chemicals and preparation

Information on the chemicals used and preparations made are provided in **Supplementary Text S1**.

### 2.3 Fish maintenance, generation, and identification of germ-free zebrafish at larval and juvenile stage

Wild-type AB zebrafish were maintained in filtered water at 28 ± 0.5 °C under a 14 h light/10 h dark cycle and were fed newly hatched *Artemia* sp. twice daily. Five groups were designated: CR, conventionally raised fish; GF, germ-free fish; ex-GF, GF fish recolonized with a conventional microbial community; GF-A, GF fish mono-associated with *Aeromonas* sp.; and GF-V, GF fish mono-associated with *Vibrio* sp. GF fish were generated using our optimized protocols and various identification methods (**Supplementary Text S2**) (42). GF zebrafish were generated with treatment by antibiotic germ-free zebrafish medium (AB-GZM) solution, povidone-iodine (PVP-I), and sodium hypochlorite (NaClO). Culture medium was renewed daily, and sterile food was provided from larvae to juvenile stage. Identification was performed using the spread plate of Trypticase Soy Agar (TSA) and blood TSA, tubes of Brain Heart Infusion Broth (BHI) medium and Trypticase Soy Broth (TSB) medium under aerobic and anaerobic conditions, and molecular techniques. For ex-GF recolonization, half of the culture water from the contemporaneous CR group was added to each ex-GF well at 5 dpf, and sterile water was added to the final volume. Thereafter, ex-GF medium was renewed daily using culture water handled in parallel with the CR group. *Aeromonas* sp. and *Vibrio* sp. were isolates previously recovered and identified from adult zebrafish intestinal microbiota in our laboratory (38). At 5 dpf, GF-A and GF-V fish were infected with 10^6^ CFU/mL of the corresponding freshly cultured bacteria in 7 mL per well (eight fish per well) for 24 h. At 6 dpf, the inoculum was replaced with sterile medium, and the fish were maintained under sterile conditions until sampling.

### 2.4 Developmental and behavioral assessment of CR, GF, ex-GF, and mono-associated fish

Hatching rate, survival rate, body length, body weight, heart rate, and locomotor behavior were evaluated to determine developmental delay from larval to juvenile stages, based on established key endpoints (summarized in **Table 1**) (43–46). Each treatment group was established in at least three six-well plates, with eight fish per well; the average hatching and survival rates were calculated with replicate groups, whereas individual fish larvae were measured for continuous developmental endpoints. For histological processing, three replicate fish per group were scored, and the scores were considered with these biological replicates. After treatment, fish were washed, anesthetized with 100 mg/L MS-222, and sampled. Samples were fixed in 4% formaldehyde for H&E and AB-PAS staining and analyzed using K-Viewer version 2.7.2.0 (47). The intestinal ultrastructure was examined by transmission electron microscopy (TEM) after fixation in 2.5% glutaraldehyde (**Supplementary Text S3**) (40). Meanwhile, the behavioral ability of fish from all groups (n=6 fish total/group) were randomly selected and was analyzed by Etho Vision XT^®^ (Noldus Information Technology, Netherlands) (**Supplementary Text S4**). Finally, the larvae samples were stored at -80°C for the subsequent transcriptomic and gene expression and metabolites assay.

**Table 1:** Summary of key endpoints for identifying delayed development of zebrafish at early life stages.

Endpoints	Zebrafish	Meaning	References
Hatching rate	Inhibited/delayed	Could be a result of decrease movement activity due to somatic abnormalities	(43)
Body length; Overall size	Shorter	may arise from skeletal defects, irregularities in somatic boundaries and disorganized muscle fibers	(43)
Body weight	Lower	Growth retardation	-–
Lethality	Higher	Exposure toxicity	(43)
Survival rate	Reduced	Important endpoints	(44)
Malformation rate	Increased	Abnormal developed yolk sac, heart	(43, 44)
Heart rate	Impaired	An important framework in small fish model; Blood flow/circulation	(43, 45)
Swim bladder	Uninflated	Secondary effects of the curved spine/muscle/skeletal phenotype, a delay in hatching that didn’t allow inflation to occur on time; reduced movement as a result of somatic defects	(43)
Behavior; Swimming; Light-dark stimulation	Reduced distance, speed,	Zebrafish cannot swim freely and feed independently	(45)
Histopathological indexes; Morphological observation	Abnormal	Corresponding to tissue/organs development	-–
Hormones	Disruption	Promote growth	(44)
Signaling pathways	Irregular	Regulation abnormal developmental stages	(46)
Transcriptomics profile	Changes	System response; Gene biomarkers	(46)

### 2.5 Differences in transcriptomic profile of GF and CR zebrafish at 7 and 14 dpf

Total RNA was extracted from three independent biological pools per group (n = 30 fish per pool), and libraries were sequenced as paired-end reads on an Illumina platform (**Supplementary Text S5**). After quality filtering, clean reads were aligned to the reference genome and were generated for differential expression analysis. DESeq2, enrichment, GO, KEGG, GSEA, and PPI with fdr_bh (Benjamini/Hochberg) adjusted *P* < 0.05 and |log2 fold change| >= 2 were considered differentially expressed (data processing and enrichment details were provided in **Supplementary Text S6**). The deposited raw data and accession links are described in the Data Availability statement.

### 2.6 Composition of microbiota in larval and juvenile CR and ex-GF zebrafish

To illustrate the critical role of microbial intervention during development, the composition differences in CR and ex-GF zebrafish at different life stages was detected and analyzed *via* the 16S rRNA high-throughput sequencing with the total DNA extracted from whole-body samples (Analysis details shown in **Supplementary Text S7**).

### 2.7 Measurement of whole-body contents of gut-brain axis hormones in zebrafish by ELISA kits

To evaluate the changes in key gut-brain axis hormones, the whole-body contents of serotonin (5-HT), glucagon-like peptide (GLP-1), cholecystokinin (CCK), peptide tyrosine-tyrosine (PYY), ghrelin, leptin (LEP), growth hormone (GH), and thyroid hormones (THs, T3, T4), as well as metabolic products (short-chain fatty acids, SCFAs) in both larvae and juvenile zebrafish were measured according to the ELISA kits directions (Protocols in **Supplementary Text S8**). Absorbance was recorded at OD 450 nm, and contents were calculated from assay-specific standard curves. Thirty fish were pooled as a sample, and three replicate biological samples per group were detected twice to contribute to six plotted assay values per analyte (n=6) of whole-body assay measurements. Hormone concentrations were detected in ng/mL or pg/mL as indicated on the figure axes, and total SCFAs presented in nmol/mL.

### 2.8 Detection of key genes in pathways related to the gut–brain axis and fish development

Total RNA was extracted from three independent pools per group (n=30 fish per sample) using RNAiso Plus reagent according to the manufacturer’s instructions (Takara Bio). RNA quality and purity were assessed before cDNA synthesis using the PrimeScript RT Reagent Kit with gDNA Eraser (Takara Bio). qRT-PCR was performed on an ABI 7300 system with a SYBR Green qPCR kit (Biosharp), and the zebrafish primers are listed in **Table 2**. The cycling program comprised 95 °C for 2 min, followed by 40 cycles of 95 °C for 15 s, 60 °C for 20 s, and 72 °C for 30 s; melt-curve analysis was then performed at 95 °C for 15 s, 60 °C for 1 min, and 95 °C for 15 s. Technical replicate reactions were averaged for each biological pooled sample. Relative expression was calculated using the 2−ΔΔCT method with *gapdh* as the reference gene.

**Table 2:** Primers of zebrafish used in this study.

Gene name	Access No.	Sequence (5 ‘-3’)	Product length (bp)
*gapdh*	NM_001115114.1	F: TCCTGAGCTCAATGGCAAGC R: GGCAGGTTTCTCAAGACGGA	97
*tg*	NM_001329865.1	F: TTTGAGGACGGTCGCTTCAG R: GCTGGCAGGTGGTTAAACAATAA	136
*ttr*	NM 001005598.2	F: GATTGCCAGTGGGAAAGTGG R: GTGGTGTACGAGAAAGGGCT	225
*tshr*	NM_001145763	F: GGCCAATGCTCCTTGATGTG R: TTGAGGGCCCAAACATTACG	103
*dio1*	NM_001007283.2	F: GCAAAGTTATCTATCAGGGAGGC R: AGAAAATGAGTCCAGGCGCT	154
*gh*	NM_001020492.2	F: CTTGGACAAAATGGCTAGAGCA R: GTTGCAGAACGACAGAGGGA	226
*crh*	NM 001007379.1	F: ACTCCAGCCTTGCTTTGAAGA R: GTGGCGAGGAGAATCTGTGC	130
*igf1*	NM_131825.2	F: TCCCAGGACACCAAAGAAACC R: CAGTTCATTCCTCCCGCTGT	135
*ghra*	NM_001083578.1	F: CGACACGCACGACTGTTGAT R: TTGTTTGCATCTGACGCTTGA	219
*PYY-a*	NM_001164371.1	F: CAACGAAAGAGCGTTGGAGC R: CATGACGGCCATTTTCAGCA	93
*cfbl*	NM_001083858.1	F: TCGAGTGGTCCAGGTTGGTA R: GACTCAGATCTCGGCTTCGG	78
*zgc:171509*	NM_001320433.1	F: GGTTTCTGGATGGGGCGTAA R: GACCACCAGAATCCCCCTGA	179
*pcna*	NM_131404.2	F: CGACAAGGAGGATGAAGCGGTAACA R: GTACTCAACCACTAGTGGAATATCTGCGGAC	154
*mki67*	NM_001277446.2	F: GCAGTCATTCCAGCTGAAGATTT R: TTTTCTTTGGGAATGTTGGCCTC	167
*cdx1b*	NM_001098762.1	F: CTCCAATCCTCCATCAACTCTTCAG R: TTTGTTCTGGTCTTCCCTCCTGAG	122
*fabp2*	NM_131431.1	F: TCAGCACTTTCCGCACACTG R: TACCTTTCCGTTGTCCTTGC	149
*oclna*	NM_212832.2	F: TGGACCCTCAAGAGGCGAT R: ACATCTTGCGGGGAGTTCTG	172
*cldn15la*	NM_131771.2	F: GCATGTTGGGTGGTGGTTTC R: TCTGCGTTCGATTGAGCTGT	124

### 2.9 Correlation analyses of microbial intervention with the changed indexes in GF and CR zebrafish

Correlation analyses were performed among selected transcriptomic, microbial, hormonal, and developmental variables. Spearman coefficients were calculated from the normalized feature profiles used to construct the heat map. The omics and phenotypic datasets indicated the relation pattern with visualization, OPLS-DA with VIP>1, *P* value>0.05, and 95% confidence ellipses to distinct patterns of groups. The module-trait from WGCNA (weighted correlation network analysis) with Pearson correlation coefficient and holm method adjust *P* values indicated the close relationship between gene expression pattern and host phenotype, such as hormone content, and developmental indexes, which were carried out for functional prediction.

### 2.10 Statistical analysis

Statistical analyses were conducted in SPSS version 20.0, figures were produced by using GraphPad Prism 10, and violin plots presented the individual plotted values with the median and interquartile range. Normality and homogeneity of variance were assessed using the Kolmogorov-Smirnov and Levene tests, respectively. Two-group comparisons used two-sided Student’s t-tests, and multi-group comparisons used one-way ANOVA followed by Dunnett’s test against the corresponding control, as applicable. All data was presented with means ± standard error (SEM) of replicates in each group, and symbols *, **, and *** stand for *P* < 0.05, 0.01, and 0.001 indicating the significant differences between control and treated groups. Transcriptomic and microbiome differential abundance calculated the *P* values adjusted with Benjamini-Hochberg method. Microbial PCoA based on Bray-Curtis distances was interpreted descriptively. LEfSe used an LDA score threshold > 2.0. PICRUSt2 pathway comparisons used one-way ANOVA followed by Duncan’s test and were considered functionally predictive. Cross-dataset correlations, OPLS-DA, and module-trait analyses were exploratory visualizations and biomarkers.

## 3 Results

### 3.1 Fluorescent labeling and colonization of *Aeromonas* sp. and *Vibrio* sp.

In this study, germ-free (GF) zebrafish were generated and validated (**Supplementary Figure S1**) according to our published protocol (42). Strains of *Aeromonas* sp. GF-Z-2 and *Vibrio* sp. GF-Z-4 were freshly cultured in TSB at 30 °C with shaking at 150 rpm, then labeled with CM-DiI according to our previous procedure (42). Fluorescence was observed under microscope *in vivo*, and plate counts were monitored daily, which brightness and distribution can determine the colonization and duration in intestine (**Figures 1A, B**). Both strain counts increased at 8–10 dpf and decreased at 11 dpf but still remained detectable after medium replacement (**Figure 1C**).

**Figure 1:**
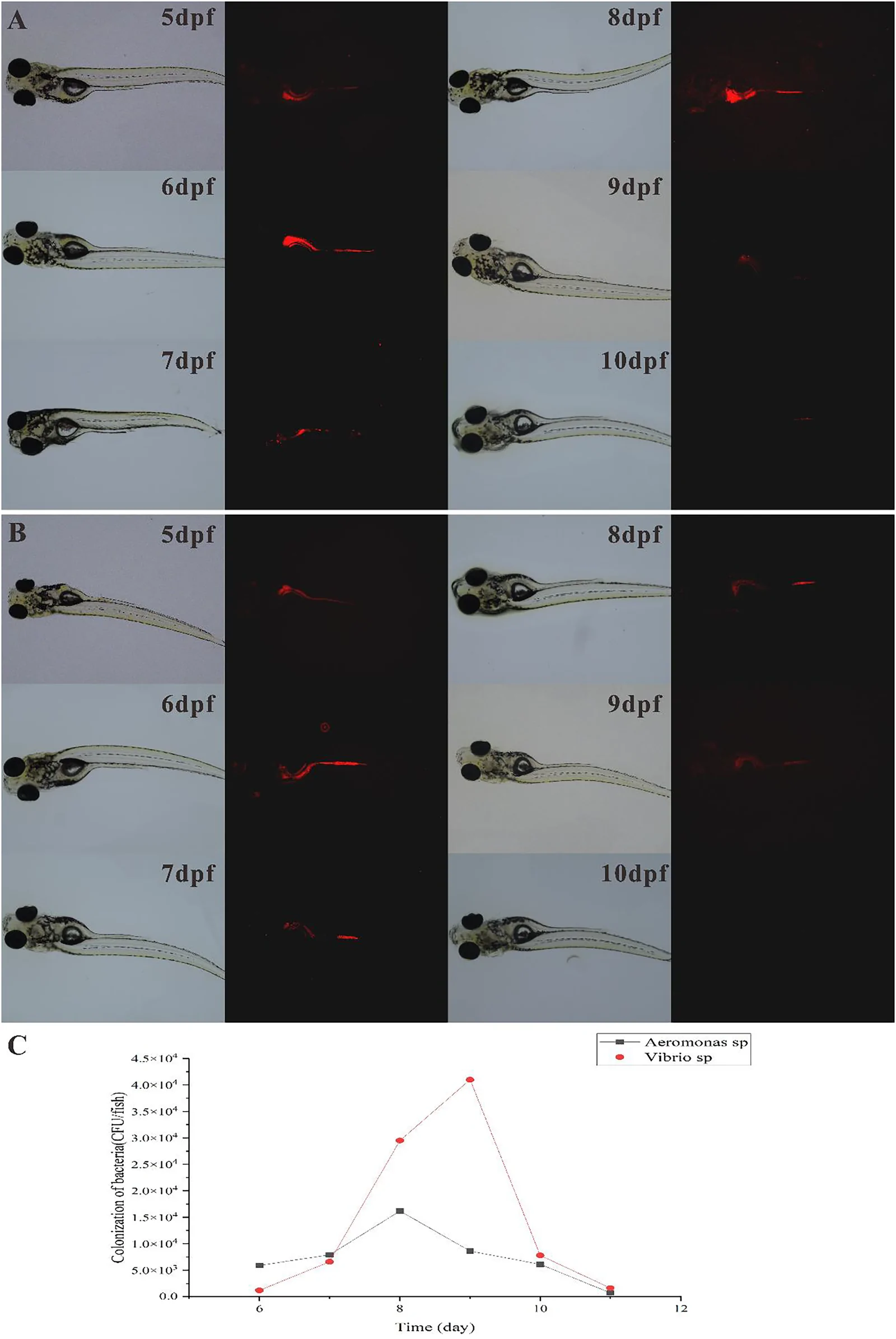
Colonization of bacterial strains in infected GF zebrafish intestines. **(A, B)** Distribution and intensity of labeled *Aeromonas* sp. and *Vibrio* sp. in GF zebrafish over time observed through fluorescence microscopy. **(C)** Number of bacteria colonized in the GF zebrafish intestines after infections.

### 3.2 Developmental indexes of various fish models at larval and juvenile stages

Developmental endpoints were recorded after establishment of the GF and microbial intervention models (**Figure 2A**). In both stages, hatching rates of CR fish at 72 hpf were 93.06% and 93.75%, while the other groups showed modest reductions (**Figure 2B**). The average survival rate of larvae showed no changes with a range of 87.7%-91.0%, but the survival of GF-14d, and related treatments were decreased significantly with 56.5%-70.22% than CR-14d (79%), respectively (**Figure 2C**). Compared with the average body length of CR-7d fish as 4045.49 μm, the GF and related groups were all significantly decreased with range of 3752.6 μm to 3965.94 μm. Similarly, compared with the body length of CR-14d groups as 4299.99 μm, the ex-GF (4106.53 μm), GF-A group (4008.12 μm), and GF-V (4029.24 μm) were significantly decreased with *P* < 0.01 (**Figure 2D**). Larval body weight ranged from 0.4838 to 0.5079 mg without significant differences, except in GF-V fish, which weighed 0.6239 mg (*P* < 0.01). In contrast, the body weight of GF-A-14d and GF-V-14d groups, at 0.3609 mg and 0.3442 mg, showed significantly decreased (*P* < 0.05) (**Figure 2E**). Heart rates in GF-7d and the corresponding microbial intervention groups ranged from 20.0 to 21.6 beats per 10 s, compared with 22.8 beats per 10 s in CR-7d fish (*P* < 0.01). Interestingly, the heart rate (beats/10s) of 14d groups ranged from 29.6-31.1 without changes, except for GF fish which showed significant decreases (*P* < 0.05) (**Figure 2F**).

**Figure 2:**
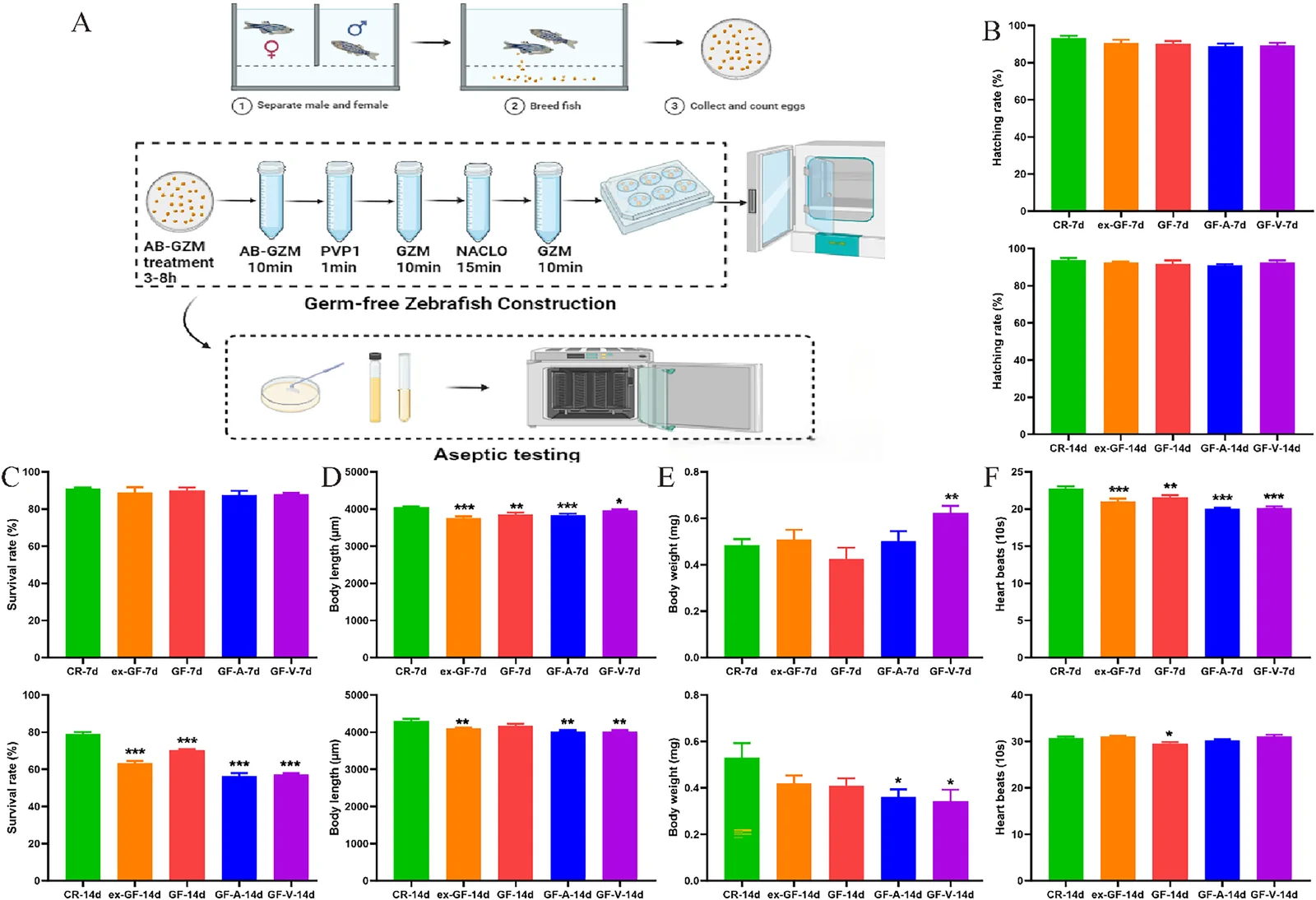
Developmental indexes of CR, ex-GF, GF, GF-A, and GF-V zebrafish larvae and juveniles. **(A)** Flow chart of GF zebrafish model generation and validation. **(B)** Hatching rate (%) at 72 hpf. **(C)** Survival rate (%). **(D)** Body length (μm). **(E)** Body weight (mg). **(F)** Heart beats (10s) of zebrafish in 7d and 14d groups. Each group was established in three six-well plates with approximately eight fish per well for recording and calculation. Bars show mean ± SEM, and symbols of *, **, *** stand for *P* < 0.05, 0.01, 0.001, indicating the significant differences between treated groups and corresponding control groups.

### 3.3 Behavioral ability of CR, ex-GF, GF, and mono-associated fish

Behavioral analyses included trajectory plots, heatmaps, total distance traveled, movement duration, velocity, high-velocity (“manic”) bout frequency and duration, active/inactive swimming durations, and a 25-min light–dark transition test (**Figure 3**). Trajectory plots and heat maps indicated the microbial intervention groups moved less toward the center area and preferred to swim along the walls and displayed significantly decreased behavioral ability (**Figures 3A, B**). The moving distance, moving time, and average velocity of ex-GF, GF, GF-A, and GF-V groups were significantly decreased (**Figures 3C–E**). Compared with manic frequency and cumulative manic time of CR-7d fish, the GF-A and GF-V groups were significantly decreased (*P* < 0.05), respectively (**Figures 3F, G**). The active frequency, cumulative active time, and static frequency of GF, GF-A, and GF-V fish decreased significantly (*P* < 0.001) (**Figures 3H–J**). Notably, the cumulative static duration of CR fish was 812.68s, the GF group 910.33s, the GF-A group (934.32s) was increased (*P* < 0.05), and GF-A (921.36s) and GF-V groups increased significantly (*P* < 0.001) (**Figure 3K**). In the light-dark test, the movement velocity of treated fish all decreased under light stimulation compared with the CR groups. When dark, the velocity decreased compared with those stimulated by light, and the velocity of the ex-GF group increased, while the move speeds of GF and bacterial infected groups were still obviously lower than CR-7d fish (**Figure 3L**). When light, the CR and ex-GF fish larvae also displayed higher sensitivity in response to environmental changes, than the GF and bacterial infection models.

**Figure 3:**
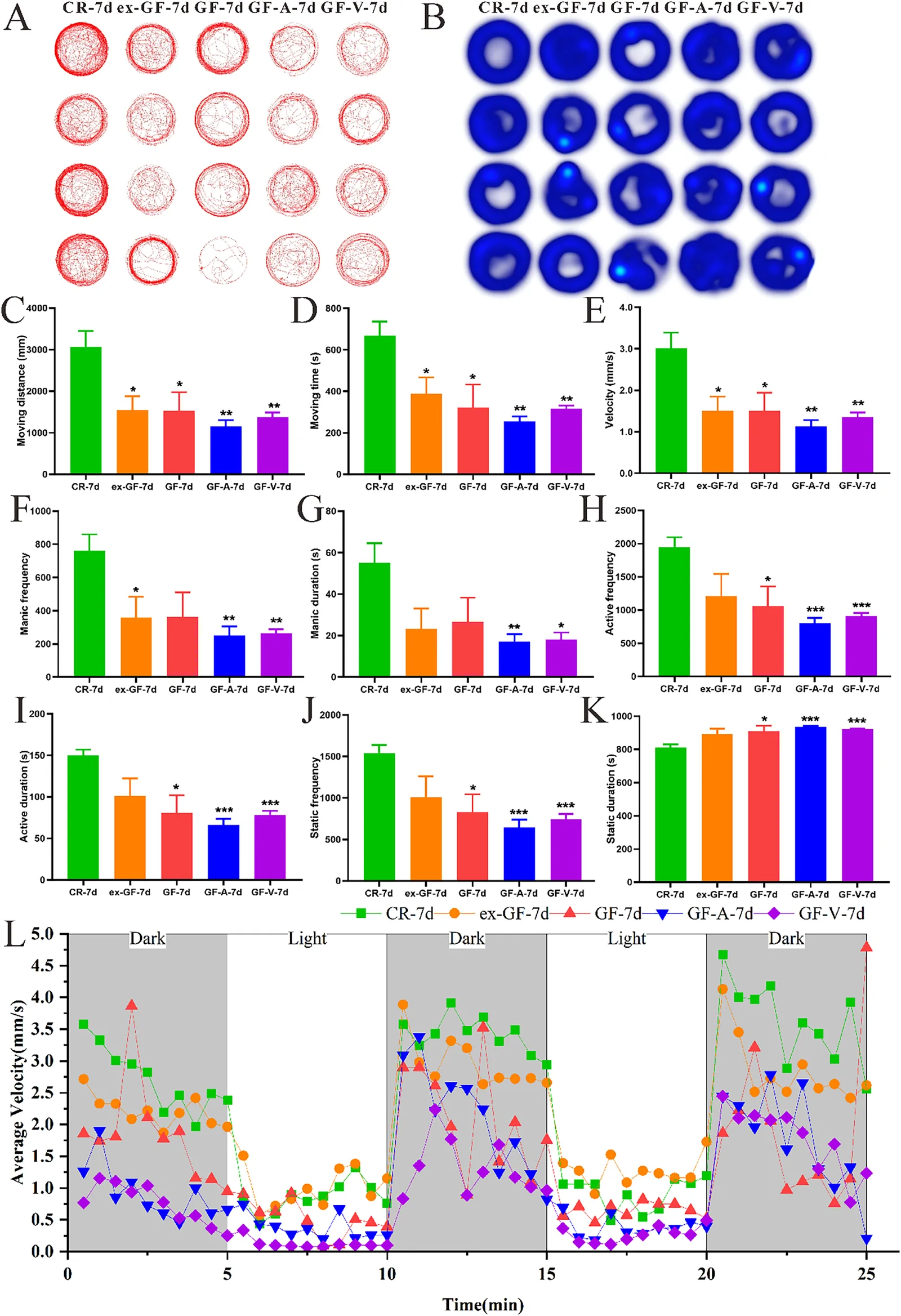
Behavioral ability of CR, ex-GF, GF, GF-A, and GF-V zebrafish at 7 dpf. **(A)** Trajectories. **(B)** Heat maps. **(C)** Moving distance (mm). **(D)** Moving time (s). **(E)** Velocity (mm/s). **(F)** Manic frequency. **(G)** Manic duration (s). **(H)** Active frequency. **(I)** Active duration (s). **(J)** Static frequency. **(K)** Static duration (s). **(L)** 25-minute light-dark stimulation. Six fish per group were recorded and included in the quantitative analysis (n=6) with mean ± SEM. Symbols of *, **, *** stand for *P* < 0.05, 0.01, and 0.001, indicating the significant differences by one-way ANOVA with Dunnett’s test of treated groups against control.

At 14 dpf, trajectory plots and heat maps showed less frequent movement toward the center and greater wall preference in ex-GF, GF, GF-A, and GF-V fish (**Figures 4A, B**). The moving distance, moving time, and average velocity of ex-GF, GF, GF-A, and GF-V groups all showed dramatical decreases (*P* < 0.05, 0.01, 0.001) compared with CR group (**Figures 4C–E**). Manic frequency and duration, active frequency and duration, and static frequency were noticeably lower in the microbial-depleted and microbial-colonized groups (**Figures 4F–J**). Static duration was increased in ex-GF (533.07 s), GF (538.49 s), and GF-A fish (553.52 s) relative to CR fish (*P* < 0.05; **Figure 4K**). In the light-dark test, CR fish maintained a comparatively stable response, whereas the microbial-intervention groups, particularly GF-A-14d and GF-V-14d fish, showed greater variability (**Figure 4L**). GF fish showed the weakest velocity response overall, indicating the association between microbial depletion and reduced behavioral ability during development.

**Figure 4:**
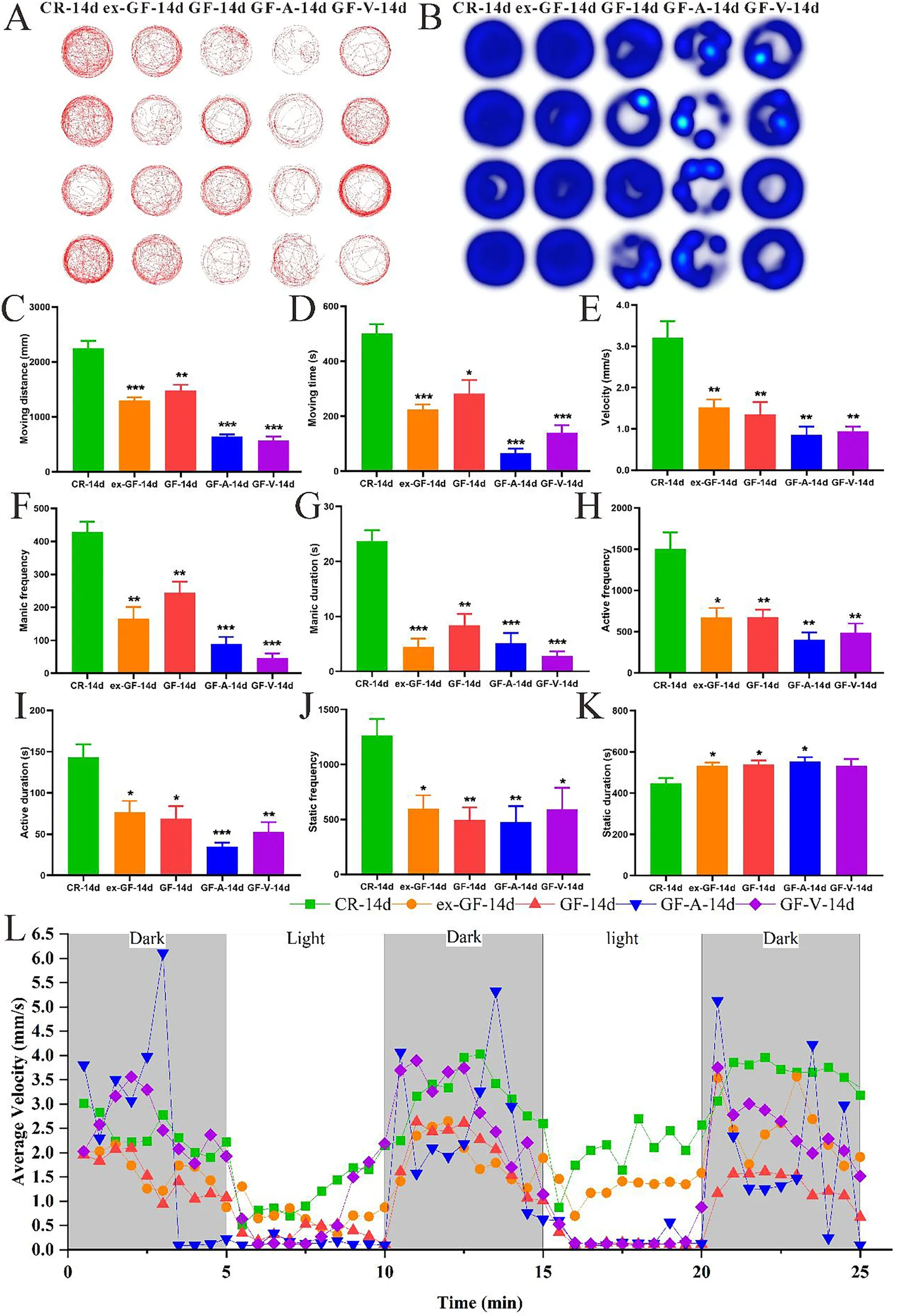
Behavioral ability of CR, ex-GF, GF, GF-A, and GF-V zebrafish at 14 dpf. **(A)** Trajectories. **(B)** Heat maps. **(C)** Moving distance (mm). **(D)** Moving time (s). **(E)** Velocity (mm/s). **(F)** Manic frequency. **(G)** Manic duration (s). **(H)** Active frequency. **(I)** Active duration (s). **(J)** Static frequency. **(K)** Static duration (s). **(L)** 25-minute light-dark stimulation. Six fish per group were recorded and included in the quantitative analysis (n=6) with mean ± SEM. Symbols of *, **, and *** stand for *P* < 0.05, 0.01, and 0.001, indicating the significant differences by one-way ANOVA with Dunnett’s test of treated groups against control.

### 3.4 Histopathological analysis and ultra-structure of CR and GF fish intestines

Histopathological analysis revealed intestinal defects in both larval (**Figures 5A–E**) and juvenile (**Figures 5F–J**) GF zebrafish. A disruption to intestinal homeostasis involving impaired epithelial integrity and changed clefts, reduced gut thickness, and distribution of goblet cells appeared in the middle to posterior gut in the GF-7d and GF-14d groups. Based on the above indexes and in reference to our previous studies (40, 47, 48), the histopathological score was comprehensively calculated (**Figure 5K**). GF fish showed serious damage or abnormal development, with significantly higher score than CR larvae and juveniles, while the recolonized or bacterial infected fish showed better than GF fish.

**Figure 5:**
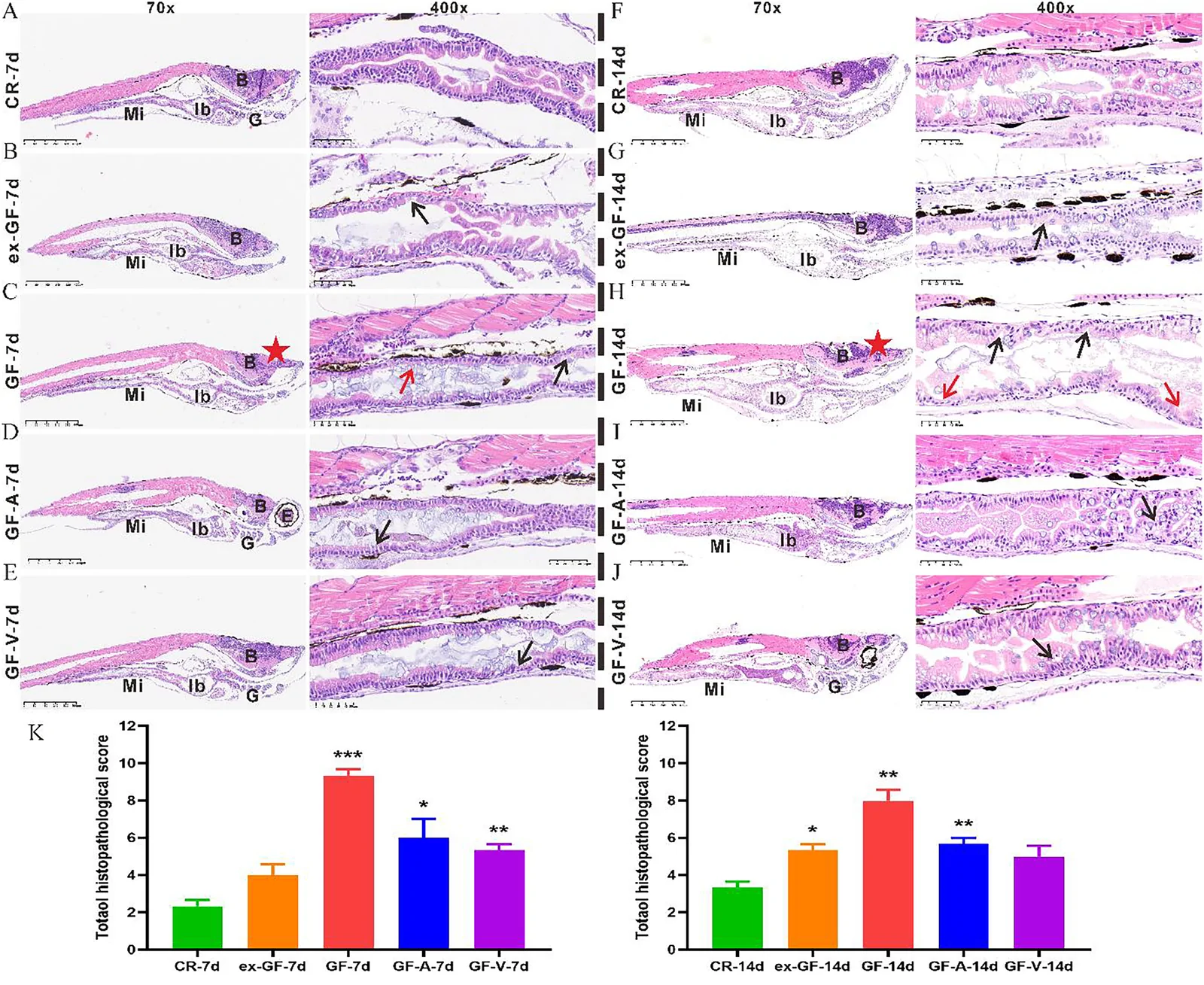
Histopathological analysis of CR, ex-GF, GF, GF-A, and GF-V zebrafish intestines with H&E staining. Whole-body (70×) view and magnifying images of intestines (400×) in CR-7d **(A)**, ex-GF-7d **(B)**, GF-7d **(C)**, GF-A-7d **(D)**, and GF-V-7d **(E)** larval zebrafish (n=3 replicates/group) showed disruption and abnormal structure. Meanwhile, whole-body (70×) view and magnifying images of intestines (400×) in CR-14d **(F)**, ex-GF-14d **(G)**, GF-14d **(H)**, GF-A-14d **(I)**, and GF-V-14d **(J)** juvenile zebrafish (n=3 replicates/group). Impaired epithelial integrity and changed clefts (black arrow), reduced gut thickness (red arrow), and distribution of goblet cells appeared in middle to posterior gut especially in the GF-7d and GF-14d groups. **(K)** Based on the above indexes, histopathological score of each group was comprehensively analyzed with mean ± SEM and *P* < 0.05 indicating the significant differences. Middle intestines (Mi), Intestinal bulb (Ib), Mucus layer (ML), Brain (B, abnormal in red star), Eye **(E)**, and Gill **(G)**. The scale bars were 400 μm for 70× images and 50 μm for 400× images. Symbols of *, **, *** stand for P<0.05, 0.01, 0.001, indicating the significant differences between treated groups against control.

AB-PAS staining showed differences in the number and distribution of goblet cells and mucus layer in larvae (**Figures 6A–E**) and juveniles (**Figures 6F–J**). In the middle gut, goblet cells in GF groups were significantly less than in CR groups in both early life and juvenile (**Figure 6K**). While microbial-infected fish showed more than GF fish but still did not recover to the normal quantity. As key intestinal cells, the goblet cells influenced the secretion and production of mucus layers, could the reduction of which could impact the digestive and absorption functions in host.

**Figure 6:**
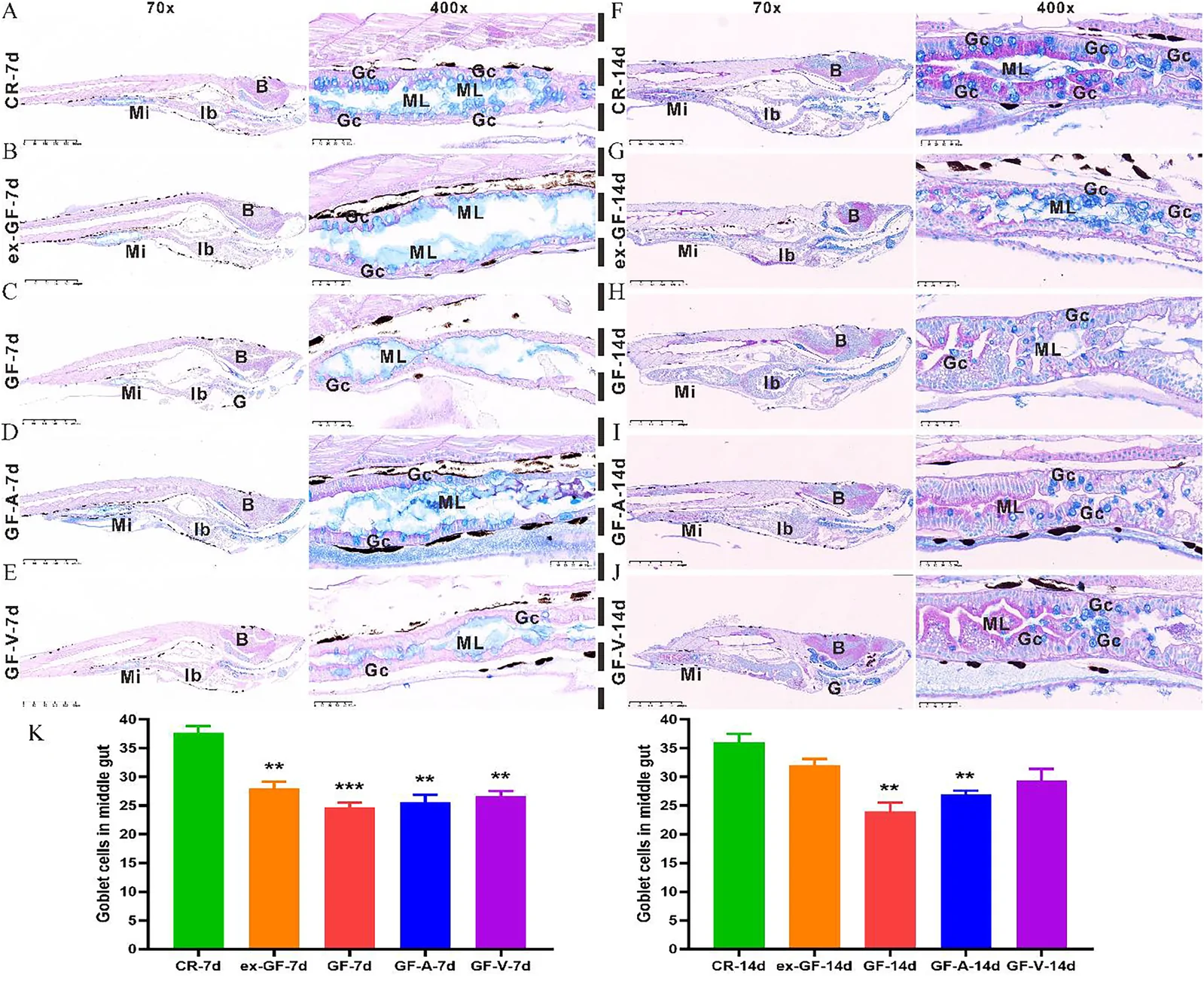
Histopathological analysis of CR, ex-GF, GF, GF-A, and GF-V zebrafish intestines with AB-PAS staining. The whole-body (70×) view and magnifying images of intestines (400×) in CR-7d **(A)**, ex-GF-7d **(B)**, GF-7d **(C)**, GF-A-7d **(D)**, and GF-V-7d **(E)** larval zebrafish (n=3 replicates/group), which showed the number and distribution of goblet cells and the mucus layer in intestines under magnification images (400×, correspondingly to A-E). Meanwhile, the whole-body (70×) view and magnifying images of intestines (400×) in CR-14d **(F)**, ex-GF-14d **(G)**, GF-14d **(H)**, GF-A-14d **(I)**, and GF-V-14d **(J)** juvenile zebrafish (n=3 replicates/group). **(K)** Based on the above indexes, the histopathological score of each group was comprehensively analyzed with mean ± SEM and *P* < 0.05 indicating the significant differences. Middle intestines (Mi), Intestinal bulb (Ib), Mucus layer (ML), Brain (B, abnormal in red star), Goblet cells (Gc), and Gill **(G)**. The scale bars were 400 μm for 70× images and 50 μm for 400× images. Symbols of **, *** stand for P<0.01, 0.001, indicating the significant differences between treated groups against control.

Further, the ultra-structure of larvae appeared to disrupt intestinal homeostasis between CR-7d, ex-GF-7d, and GF-7d zebrafish, involving abnormal microvilli, mitochondria damage, and distinct broken of tight junctions under microscope (**Figures 7A–C**). Similarly, compared to the images of intestines in CR-14d and ex-GF-14d juveniles, the GF-14d zebrafish also showed obvious damages of microvilli, mitochondria and tight junction at some sites (**Figures 7D–F**). Comparison of the intestinal ultra-structure, the tight junctions, microvilli disruption/damage, intercellular space and vacuoles showed significant differences between CR, ex-GF, and GF zebrafish. In detail, the microvilli became rare and irregularly arranged in brush borders in GF fish both at 7 and 14 dpf, while CR and ex-GF were well-defined and regular. Interestingly, the tight junction between intestinal epithelial cells in GF fish were more broken than the normal ones in ex-GF and CR fish. Notably, the mitochondria in GF larvae were distorted, with some outer membrane discontinuities or missing membranes altogether, and structures of cristae were reduced or hardly visible (**Figures 7C, F**). In contrast, the mitochondria in CR larvae and juveniles showed clear cristae and double membranes.

**Figure 7:**
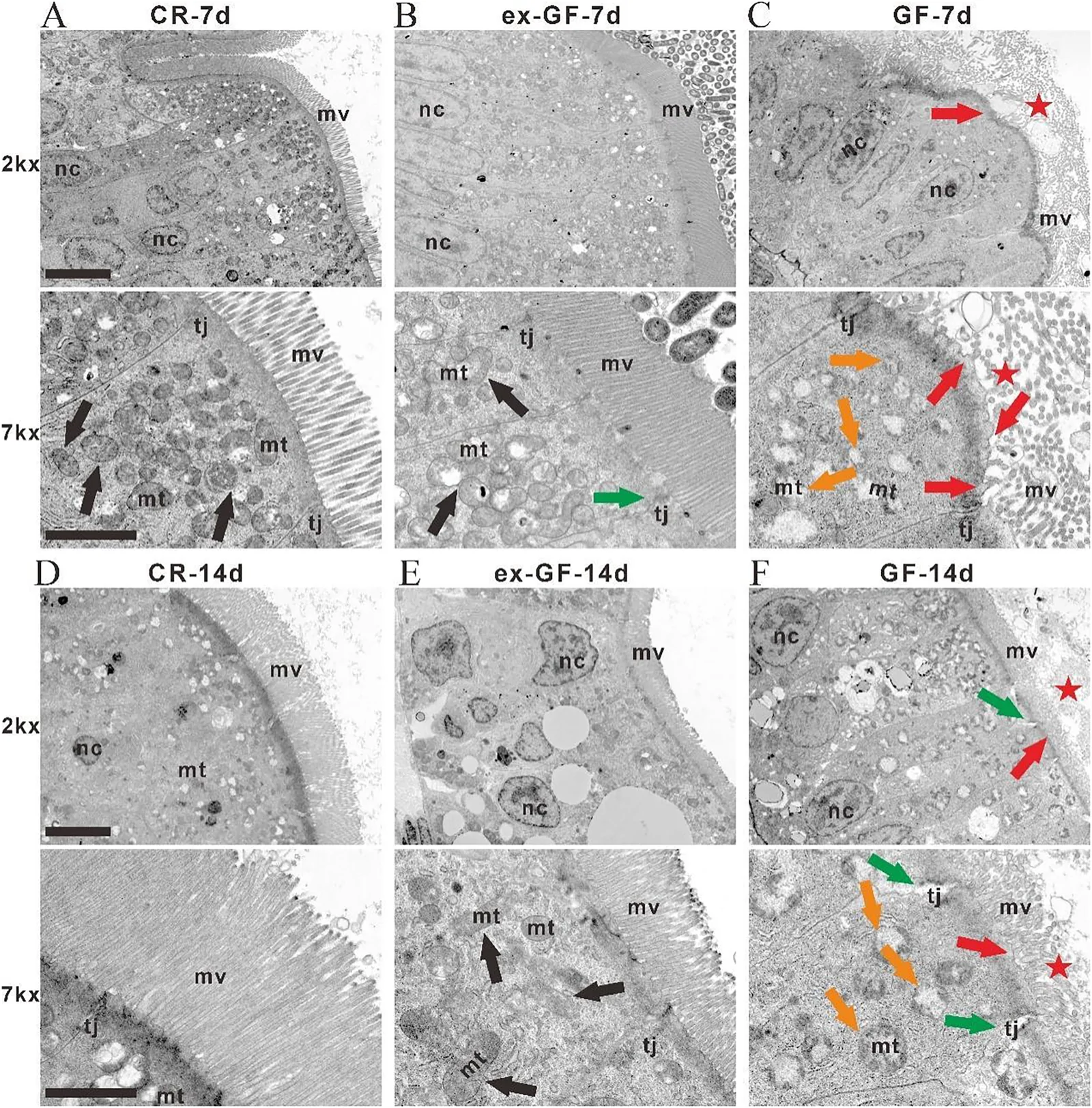
Ultra-structure of CR, ex-GF, and GF zebrafish intestines identified by TEM. Images of intestines (2.0k×) in CR-7d **(A)**, ex-GF-7d **(B)**, and GF-7d **(C)** larval zebrafish (n=30 slices/group), with the disruption of intestinal homeostasis including microvilli, mitochondria damages, and distinctly broken tight junctions at larger magnification images [7.0k×, correspondingly to **(A–C)**]. The images of intestines (2.0k×) in CR-14d **(D)**, ex-GF-14d **(E)**, and GF-14d **(F)** zebrafish (n=30 slices/group), with the disruption of intestinal homeostasis including microvilli, mitochondria damages, and distinctly broken tight junctions at larger magnification images [7.0k×, correspondingly to **(D–F)**]. Abnormal microvilli (red star), edges of epithelial cells (red arrow), and tight junctions (green arrow) appeared at some sites of the gut, especially in the GF-7d and GF-14d groups. Microvillus (mv: black arrow), nucleus (nc), mitochondria (mt: black and orange arrow) and tight junction (tj: green arrow). The scale bars were 5.0 μm for 2.0k× images and 2.0 μm for 7.0k× images.

### 3.5 Changed transcriptomic profiles in CR, ex-GF, and GF zebrafish at early life and juvenile stages

Transcriptomic profiles differed by developmental stage and microbial status (**Figure 8**). PCA separated 7- and 14-dpf samples and distinguished GF samples from their corresponding CR groups (**Figure 8A**). Across all samples, 33,223 genes were detected, and the volcano plot revealed a strong enrichment of inhibited genes, particularly significant different expression genes (DEGs). In GF-7d versus CR-7d fish, 110 genes were downregulated and 17 were upregulated at adjusted *P* < 0.05 and |log2 fold change| >= 2 (**Figure 8B**). In GF-14d versus CR-14d fish, 451 genes were downregulated and 188 were upregulated (**Figure 8C**). The larger number of DEGs at 14 dpf indicated a stronger stage-associated transcriptional difference (**Figure 8D**). Ex-GF-14d versus CR-14d fish showed 437 upregulated and 209 downregulated genes, whereas GF-14d versus ex-GF-14d fish showed 464 upregulated and 1,187 downregulated genes. At 7 dpf, 15 DEGs were unique to GF versus ex-GF, 55 were unique to GF versus CR, and 71 were shared among the three groups (**Figure 8E**). At 14 dpf, 229 DEGs were unique to GF versus CR, 339 to ex-GF versus CR, and 1,009 to GF versus ex-GF; 370 DEGs overlapped between the GF-versus-CR and GF-versus-ex-GF comparisons (**Figure 8F**). Heat maps showed increased expression of *nr1d4b*, *guca1a*, and *cdkn1a* and decreased expression of *slc2a6*, *clca1*, and *krt96* in GF-7d fish (**Figure 8G**). At 14 dpf, *lpl*, *per2*, *cry1b*, *irg1l*, and *muc13b* were more highly expressed in CR fish, whereas *dpysl4* and *tubb5* were more highly expressed in GF fish (**Figure 8H**). GSEA identified immune, inflammatory, migration, transmembrane-signaling, protein-activity, and cytokine-activity terms enriched in the GF-7d vs CR-7d comparison (**Figure 8I**). At 14 dpf, enriched terms included iron-ion binding, inflammatory and immune responses, response to bacteria, and peptidase activity (**Figure 8J**). Protein–protein interaction (PPI) networks constructed from the DEGs showed downregulation in GF-7d compared with CR-7d groups, with irg1l, mmp13a, c2, cfbl, and mpx identified as key hub genes involved in apoptosis and inflammatory pathways. In the GF-14d versus CR-14d comparison, cdk1 and mmp9 were key hub genes associated with cell cycle regulation and immune responses (**Figures 8K, L**).

**Figure 8:**
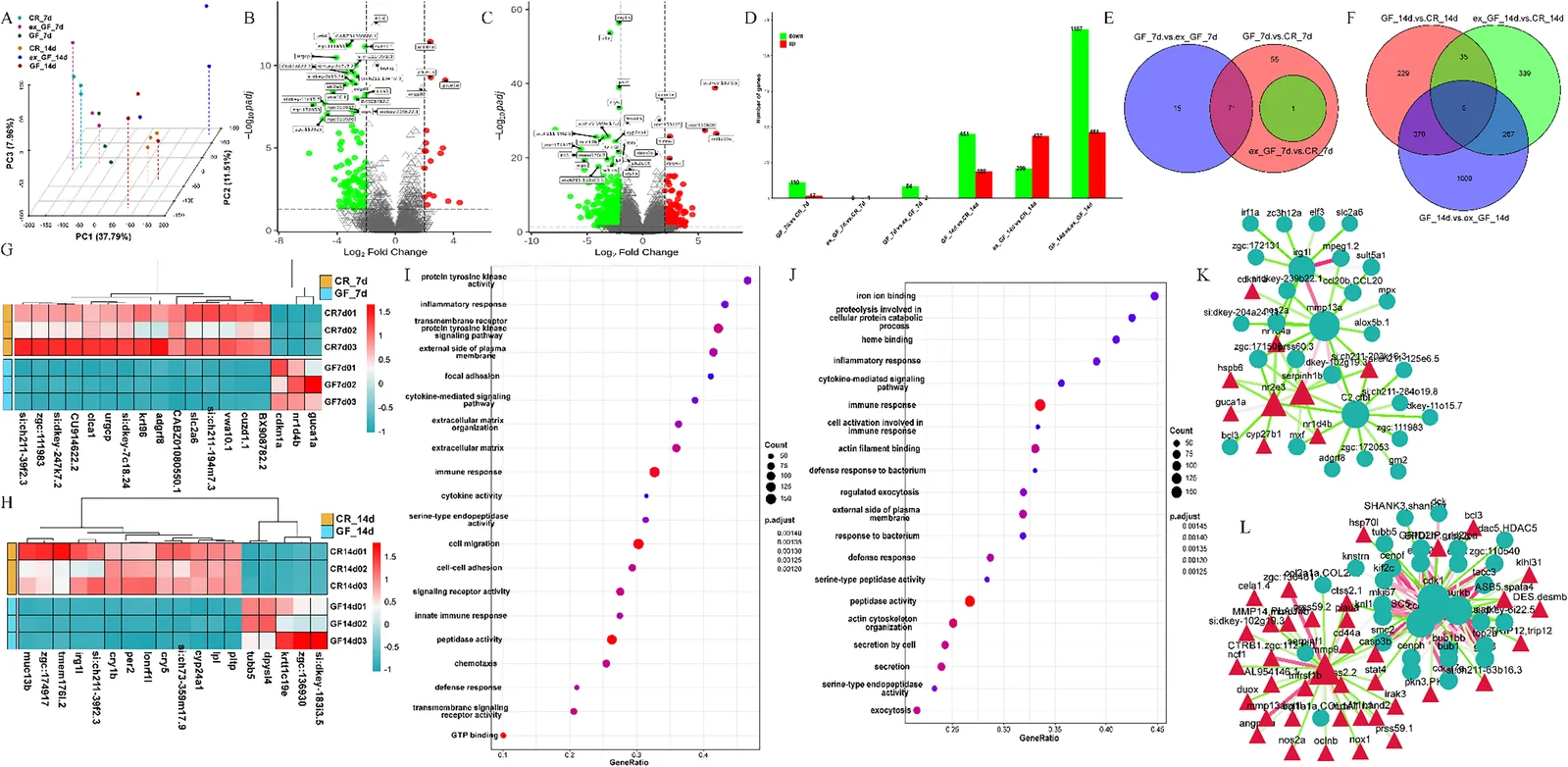
Transcriptomic profiles of CR, ex-GF, and GF zebrafish at early life and juvenile stages. **(A)** PCA enabled the visualization of differences in transcriptomic profiles of CR, GF, and ex-GF zebrafish at both life stages. **(B, C)** A Volcano Plot displayed significant DEGs in GF-7d vs CR-7d groups, and GF-14d vs CR-14d groups with name of the top 30, with |log2 fold change| > 2 and adjusted *P* < 0.05 were classified as up- (red dots) and down-regulated (green dots). **(D)** Venn and UpSet plots visualized intersections of DEG sets between treated and control groups at 7d and 14d. **(E, F)** The DEGs from three pairwise comparisons, namely GF-7d vs CR-7d, ex-GF-7d vs CR-7d, and GF-7d vs ex-GF-7d, were visualized using a Venn diagram to identify overlapping sets, as well as the 14d groups. **(G, H)** A hierarchical clustering heat-map of the top 18 DEGs was generated based on Z-score normalized expression values in GF and CR zebrafish at 7d and 14d stages. **(I, J)** Gene Set Enrichment Analysis (GSEA) was performed to identify GO pathways enriched at the extremes of the global gene expression fold-change ranking in GF-7d vs CR-7d groups and GF-14d vs CR-14d groups. **(K, L)** Protein-protein interaction network potentially reflects the functions changed between GF-7d vs CR-7d and GF-14d vs CR-14d groups. Green-down and red-up in **(K)** and Green-up and red-down in **(L)**.

### 3.6 Composition of microbiota in CR and ex-GF zebrafish at early life stages

The gut microbiota composition of CR and ex-GF zebrafish at early life stages was analyzed to identify dominant taxa and their functional roles in the host (**Figure 9**). The 16S rRNA gene dataset comprised three replicate samples per group. After filtering, merging, chimera removal, and DADA2 denoising, taxonomic profiles were summarized at the ASV and genus levels (Raw reads deposited as FASTQ files). The α diversity analyses showed no changes in the indexes among those groups with Wilcox test, however, in β diversity analyses, the dominated phyla in larvae and juvenile were Proteobacteria in all groups ranged with >66.9% abundance, and Actinobacteriota was significantly enriched in juveniles (6.92~19.5%), while the abundance of Myxococcota was higher in larvae. The abundance of Bacteroidota, Firmicutes, and Chloroflexota phyla was higher in ex-GF fish than corresponding CR groups at both stages (**Figure 9A**). Moreover, the heat-map of bacteria with hierarchical clustering revealed distinct group- and stage-associated microbial patterns. Temporal effects were pronounced in terms of *Pseudomonas*, *Mycobacterium*, and *Aeromonas*, which exhibited near-exclusive dominance in CR-14d groups, while *Thermosulfidibacter*, *Zavarzinia*, *Limnobacter*, and *Acidovorax* were enriched in CR-7d and ex-GF-7d groups (**Figure 9B**).

**Figure 9:**
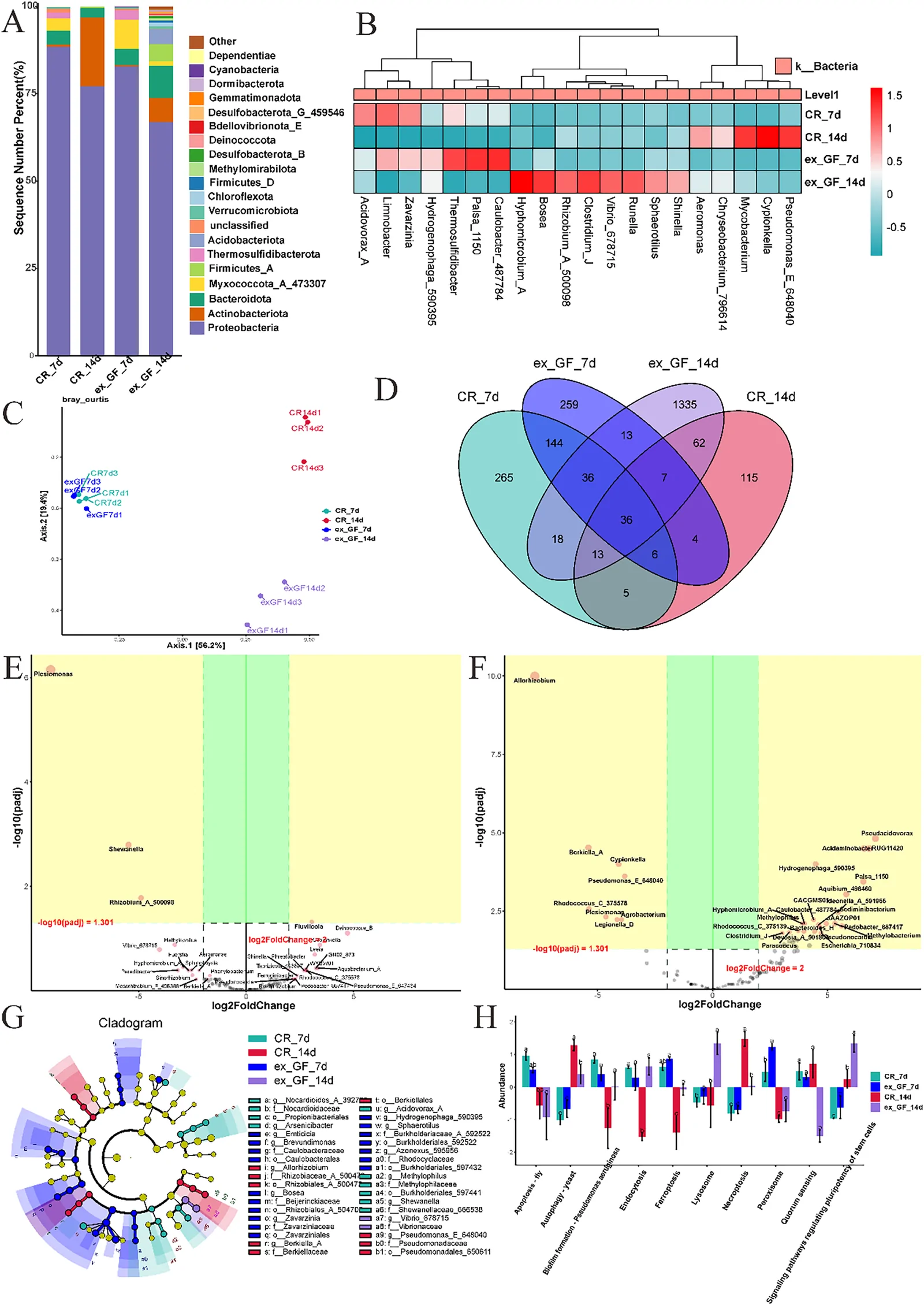
Composition of microbiota in CR and ex-GF zebrafish at early life and juvenile stages. **(A)** The average sequence number percent (%) of major phyla in CR and ex-GF zebrafish at 7d and 14d that appeared with the top 20 and “Others” was classified as <0.001%. **(B)** Heat-map visualization of the most abundant features across different groups was generated in the Basic Statistics module. **(C)** In the PCoA analysis, the 2D picture of category2 with bray-curtis distance showed the distinct distribution of gut microbiota in the four groups. **(D)** The Venn diagram presented the common, shared, and unique bacterial features among CR-7d, ex-GF-7d, CR-14d, and ex-GF-14d zebrafish. DESeq2 showed significantly changed microbial features in ex-GF-7d vs CR-7d **(E)** and ex-GF-14d vs CR-14d **(F)** groups, with the |log2 Fold change| >2 and adjusted *P* < 0.05. **(G)** The cladogram of different genera, family, and order levels between these groups were analyzed by LEfSe analysis. **(H)** Functional differences in key metabolism pathway of microbial cellular processes were analyzed using ANOVA followed by Duncan’s multiple comparison test (*P* < 0.05), such as apoptosis, lysosome, necroptosis, and signaling pathways regulating the pluripotency of stem cells changed in compared groups.

Principal coordinates analysis (PCoA) showed that Axis 1 and Axis 2 explained 56.2% and 19.4% of the total variation, respectively. Sample separation was driven primarily by developmental stage, whereas CR and ex-GF samples partially overlapped within the same stage (**Figure 9C**). The Venn diagram illustrated the distribution of microbial features across groups; for example, ex-GF-14d fish harbored the highest number of unique features (1335), followed by CR-7d (265) and CR-14d (115). There were five features shared among all four groups, indicating minimal overlap in microbial composition. Notably, the number of shared features in the CR and ex-GF groups at 7d and 14d were more than in the time-related groups, indicating that the ex-GF treatment rebuilt microbial communities (**Figure 9D**). DESeq2 analysis identified eight increased and seven decreased genera in ex-GF-7d versus CR-7d; *Plesiomonas* and *Shewanella* were depleted, whereas *Fluvicola*, *Deinococcus*, and *Aquabacterium* were enriched (**Figure 9E**). In ex-GF-14d versus CR-14d, 35 genera met the adjusted significance criteria, including enrichment of *Pseudacidovorax*, *Acidaminobacter*, and *Hydrogenophaga* and depletion of *Allorhizobium*, *Berkella*, *Pseudomonas*, and *Rhodococcus* (**Figure 9F**). The LEfSe (linear discriminant analysis effect size) analysis highlighted biomarkers significantly enriched in specific conditions, which revealed distinct enriched bacteria. Notably, a cladogram was generated based on linear discriminant analysis (LDA, scores >2.0): *Vibrio* and *Burkholderiales* were significantly enriched in the ex-GF-7d and ex-GF-14d groups, suggesting a shift toward metabolically active or stress-responsive communities under experimental treatment. In contrast, the CR groups showed greater stability over time, with enrichment of early colonizers such as *Pseudomonadales*. These findings indicated that the experimental intervention induced significant microbial reorganization, particularly at the later time point (**Figure 9G**). Key functional pathways exhibited changes between fish at different stages (*P* < 0.05), such as Apoptosis and Autophagy. Biofilm formation was markedly enriched in ex-GF-14d, while necroptosis peaked in CR-14d. The stem cell signaling pathways showed exclusive activation in CR-14d and ex-GF-14d, indicating time-exogenous interactions were prominent from 7d to 14d in fish and amplified functional shifts (**Figure 9H**).

### 3.7 Contents of key gut-brain axis hormones and metabolites measured by ELISA kits

Whole-body concentrations of hormones and total SCFAs differed among CR, ex-GF, GF, GF-A, and GF-V fish at 7 and 14 dpf (**Figure 10**). GH, T3, T4, and 5-HT concentrations were lower in the microbial-depleted and -colonized groups than in the corresponding CR groups at both stages (*P* < 0.001), except for T4 in GF-14d fish (**Figures 10A–D**). Moreover, the levels of major gut-brain hormones like GLP-1, CKK, and PYY in ex-GF, GF, GF-A, and GF-V groups at both 7d and 14d were all dramatically inhibited (*P* < 0.001) compared to CR fish (**Figures 10E–G**). Ghrelin was decreased in ex-GF-7d and GF-V-7d fish but did not differ significantly in GF-7d or GF-A-7d fish (**Figure 10H**). Leptin showed a similar significant decrease (*P* < 0.001; **Figure 10I**). Total SCFA content ranged from 12.66 to 22.09 nmol/mL in the microbial intervention groups compared with 23.25 and 24.29 nmol/mL in the corresponding CR groups (**Figure 10J**).

**Figure 10:**
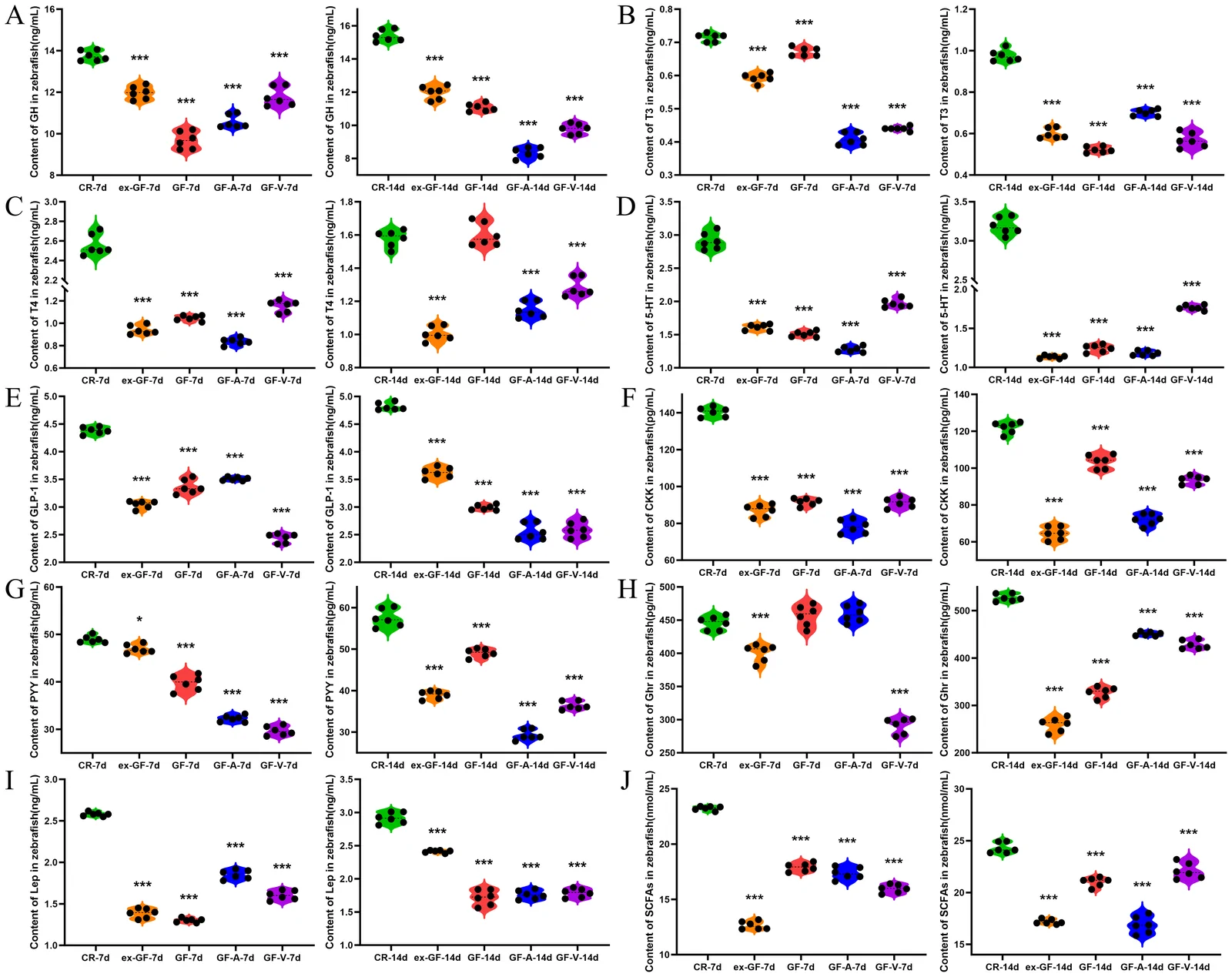
Contents of gut-brain axis hormones and SCFAs metabolites in whole-body zebrafish larvae and juveniles. In both stages, the contents of crucial gut-brain axis hormones, including developmentally related GH **(A)**, T3 **(B)**, T4 **(C)**, 5-HT **(D)**, GLP-1 **(E)**, CKK **(F)**, PYY **(G)**, ghrelin **(H)**, and leptin **(I)**, were detected by ELISA kits from the whole-body zebrafish. The key metabolites of SCFAs **(J)** were also measured. Thirty fish were pooled as one biological sample, and three replicate samples per group were detected twice (n=6 values/group). Hormone concentrations were detected with ng/mL or pg/mL levels and total SCFAs with nmol/mL. Violin plots show individual values with the median and interquartile range. Symbols of *, *** stand for P < 0.05, 0.001 by one-way ANOVA with Tukey’s multiple comparisons test.

### 3.8 Correlation analyses of microbial intervention and expression of key genes in gut-brain axis and fish development

The key genes of promoting factors (*crh*, *tshβ*), thyroid hormone synthesis (*tg*), thyroid hormone transport (*ttr*), and deiodination (*dio1*) may affect fish growth, early organ development, and swimming ability (49, 50). Expression of related genes in the gut-brain axis hormones, such as thyroid regulation (*crh*, *tg*, *ttr*, *tshr*, and *dio1*), growth (*gh*, *igf1*, and *ghra*), endocrine signaling (*pyya*), and immune responses (*cfbl*, *zgc:171509*, and *mmp13a*) were quantified to evaluate the fish growth and development (**Figure 11**). In the 7 dpf larvae, the ex-GF group showed decreased expression in *tg* (*P* < 0.001), *tshr* (*P* < 0.05), *dio1* (*P* < 0.05), *gh* (*P* < 0.01), *igf1* (*P* < 0.05), *PYY-a*, *cfbl* (*P* < 0.001), *zgc:171509* (*P* < 0.001), and *mmp13a* (*P* < 0.05) but increased expression in *ttr* (*P* < 0.05), crh (*P* < 0.01), and *ghra* (*P* < 0.05), compare with the control groups, respectively (**Figure 11A**). Similarly, the GF group showed inhibition of *tg* (*P* < 0.01), *tshr* (*P* < 0.05), *dio1* (*P* < 0.001), *gh* (*P* < 0.01), *igf1*, *ghra* (*P* < 0.05), *cfbl* (*P* < 0.001), *zgc:171509* (*P* < 0.001), and *mmp13a* (*P* < 0.05), while increased expression in *ttr*, *crh*, and *PYY-a* (*P* < 0.05) compare with the control groups, respectively (**Figure 11B**). Compared with the control group, the GF-A group showed increased expression of *tg* (*P* < 0.05), *ttr*, *tshr*, *crh* (*P* < 0.05), *igf1*, *PYY-a*, and *ghra* (*P* < 0.001), while decreased expression in *dio1* (*P* < 0.001), *gh* (*P* < 0.05), *cfbl* (*P* < 0.001), and zgc:*171509* (*P* < 0.001). Compared with the control group, the GF-V group showed increased expression of *tg* (*P* < 0.001), *tshr* (*P* < 0.05), *crh* (*P* < 0.05), igf1, *PYY-a*, *ghra* (*P* < 0.01), and *mmp13a*, while decreased expression in *ttr*, *dio1* (*P* < 0.001), *gh*, *cfbl* (*P* < 0.001), and *zgc:171509* (*P* < 0.001) (**Figures 11A–C**).

**Figure 11:**
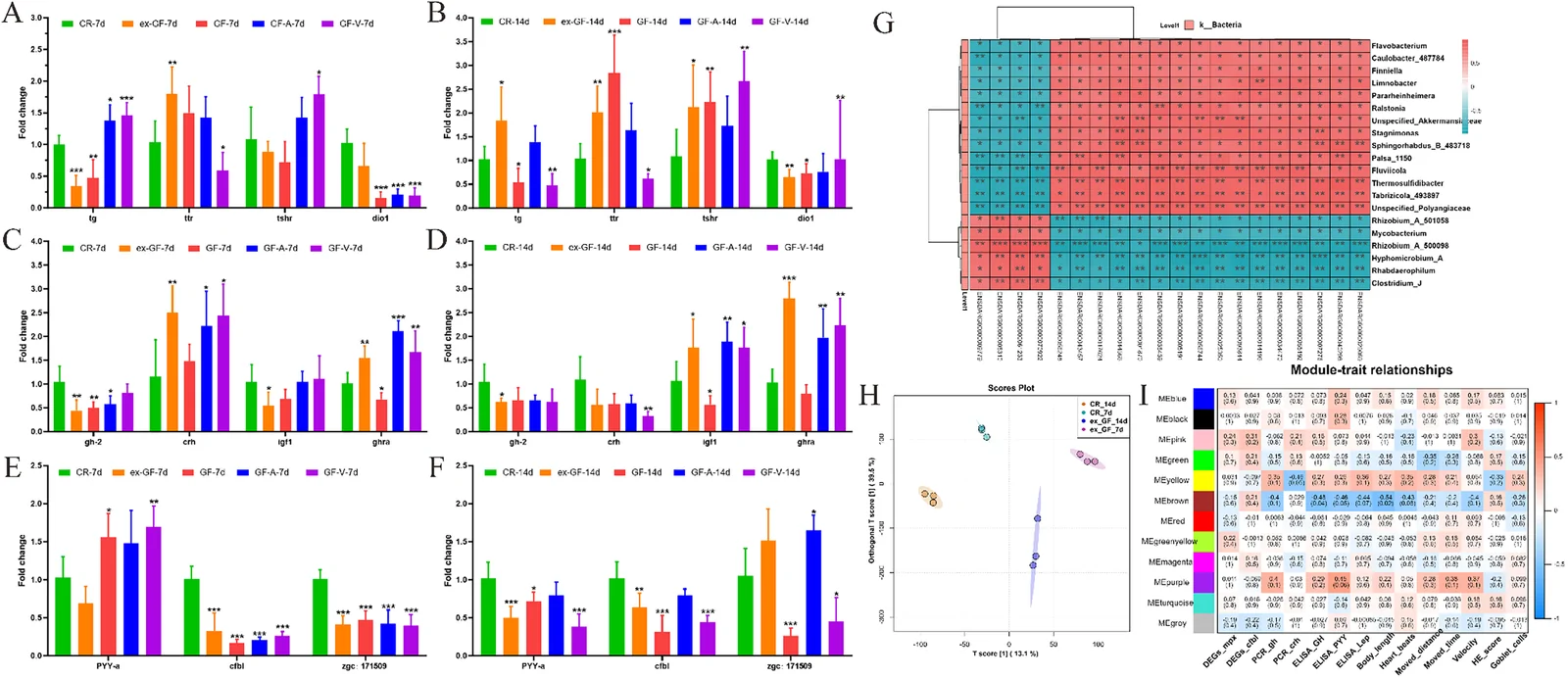
Identification of key genes’ expression and correlation analysis of DEGs with microbial intervention outcomes. Relative expression level of critical genes *tg*, *ttr*, *tshr*, and *dio1* in HPT axis in 7d **(A)** and 14d **(B)** groups. Relative expression level of genes *gh*, *crh*, *igf1*, and *ghra* in growth and developmental GH/IGF axis in 7d **(C)** and 14d **(D)** groups. Relative expression level of genes *PYY-a*, *cfbl*, and *zgc:171509* involved in hormones and DEGs immune response in 7d **(E)** and 14d **(F)** fish. The correlation analysis **(G)** of transcriptomic and microbiota in zebrafish larvae and juveniles with the top 20 DEGs and bacteria. OPLS-DA **(H)** with VIP>1, *P* value>0.05, and 95% confidence ellipses for each group showed a distinct pattern of CR-7d, ex-GF-7d, and CR-14d, ex-GF-14d groups. Module-trait relationships from WGCNA (weighted correlation network analysis) with Pearson correlation coefficient and holm method adjust *P* values **(I)** indicated the ME yellow, brown, and purple sets were significantly related to key gene expression, hormone content, and developmental indexes. Symbols of *, **, and *** stand for *P* < 0.05, 0.01, and 0.001, indicating the significant differences between treated groups and corresponding control groups.

In juveniles, the ex-GF group showed significantly increased expression of tg (P < 0.05), ttr (P < 0.01), tshr (P < 0.05), igf1 (P < 0.05), ghra (P < 0.001), and zgc:171509, along with decreased expression of dio1 (P < 0.01), gh (P < 0.05), crh and pyya (P < 0.001), and cfbl, compared with controls (**Figures 11D–F**). However, the GF group still showed decreased expression of *tg* (*P* < 0.05), *dio1* (*P* < 0.05), *gh*, *crh*, *igf1* (*P* < 0.05), *PYY-a* (*P* < 0.05), *ghra*, *cfbl* (*P* < 0.05), and zgc:*171509* (*P* < 0.05) and increased expression of *ttr* (*P* < 0.01) and *tshr* (*P* < 0.01). After bacterial infection, the GF-A group showed increased expression of *tg*, *ttr*, *tshr*, *igf1* (*P* < 0.001), *ghra* (*P* < 0.001), *cfbl*, and *zgc:171509* (*P* < 0.01), while decreased expression in *dio1*, *gh*, *crh*, and *PYY-a*. Compared with the control group, the GF-V group showed decreased expression of *tg* (*P* < 0.01), *dio1* (*P* < 0.01), *gh*, *crh*, *igf1* (*P* < 0.05), and *PYY-a* (*P* < 0.001), while increased expression was observed in *ttr* (*P* < 0.05), *tshr* (*P* < 0.001), *ghra* (*P* < 0.05), *cfbl*, and *zgc:171509*.

Correlation analyses were performed between changed transcriptomic and microbial profiles; OPLS-DA showed the distinct distribution features (**Figure 11G**). Spearman analysis was conducted with Top20 DEGs and bacteria, among which *Mycobacterium* and *Rhizobium_A* were negatively related to selected DEGs, while *Flavobacterium*, *Thermosulfidibacter* and *Limnobacter* were positively correlated between the groups (**Figure 11H**). The DEGs included *ela3l* (ENSDARG00000007276), *zgc:136930* (ENSDARG00000055192), *myl1* (ENSDARG00000014196), *fabp6* (ENSDARG00000044566), *gapdh* (ENSDARG00000043457), *si:dkey-183i3.5* (ENSDARG00000056248), *mcl1a* (ENSDARG00000009779) furtherly indicating the major bacteria in gut in regulation of key genes and signaling pathways in zebrafish growth and development. Additionally, the two gene sets in module-trait relationships analysis significantly correlated to *crh* expression, GH and PYY contents, and body length (**Figure 11I**), indicating the potential biomarkers in evaluating the effects of microbial intervention during larvae to juvenile development. Other signaling pathways with DEGs were also displayed from the enrichment analysis (**Supplementary Figure S2**). Moreover, the expanded targeted markers of cell proliferation (*pcna*, *mki67*), epithelial differentiation (*cdx1b*, *fabp2*), and intestinal barrier maturation (*oclna*, *cldn15la*) showed the inhibited trends of expression in larval GF models by both searching from zebrafish transcriptomic profile and RT-PCR detection (**Supplementary Table S1**, and **Supplementary Figure S3**). The expression of several key genes showed an increase or were gently restored at juveniles, indicating the developmental delay especially from intestinal function aspect. However, more regulatory pathways need to be verified in further studies on microbial functions in host development and immune response.

## 4 Discussion

Zebrafish harbor complex microbial communities that are commonly dominated by Proteobacteria, Firmicutes, and Fusobacteria (51, 52). However, associations of microbiota loss and recolonization with fish development remain incompletely characterized, particularly at early larval GF stages (53, 54). In this study, we combined GF, CR, recolonized ex-GF, and mono-associated zebrafish models to examine relationships among microbial status, development, behavior, intestinal morphology, transcriptomic profiles, and whole-body hormones. Multiple endpoints consistently distinguished GF from CR fish at stages with innate and adaptive immune development. The design manipulated microbial status to detect the downstream hormones, receptors, and candidate signaling pathways to establish associations and assess the therapeutic efficacy of partial phenotypic recovery.

Key basic indexes at developmental endpoints were lower in GF and bacterial-infected groups than in CR fish at 7 and 14 dpf, and ex-GF fish did not completely recover from these adverse impacts. The shorter body length, lower body weight, reduced survival rate, impaired heart rate, reduced swimming ability, and weak response to light changes proved the delay, with possible mechanism growth inhibition or organ defects. Moreover, lower survival observed at 14 dpf further indicated stage-dependent differences associated with microbial status. Locomotor behavior is an important developmental ability of zebrafish and encompasses swimming, phototaxis, hunting, feeding, and optic-flow responses (55, 56). GF and microbial-colonized groups entered the central area less frequently and showed lower movement distance, movement time, velocity, manic and active movement, together with longer static duration, than CR fish. Their velocity responses during both light and dark periods were also affected. Ex-GF fish showed partial recovery toward CR velocities, whereas GF and mono-associated fish remained more distinct, indicating the association between microbial recolonization with behavioral improvement.

As part of this study’s experimental design, intestinal morphology was evaluated as a key developmental phenotype associated with microbial colonization status. Histopathological analysis showed more pronounced intestinal morphological alterations and higher H&E scores in GF fish, whereas ex-GF and mono-associated groups showed partial structural improvement. Notably, AB-PAS staining of intestines proved that the goblet cells and mucus layer were obviously less than CR fish in GF and microbial colonized groups at innate immune stage. Similarities were observed between GF-14d and GF-A-14d juveniles regarding developing adaptive immune systems. These findings show that the rebuilding of microbiota or infected bacteria groups could improve the intestinal homeostasis of GF fish, but still do not reach to the normal level. Goblet cells control the secretion and production of mucus layers and then contribute to the intestinal digestive and absorption, protective functions in host (47). Microbiota plays key roles in intestinal maturation and function establishment (53), and the absence of them risked host health and system development, which can be identified by GF fish as the credible models to reveal the effects of microbial intervention. Moreover, intestinal folds and microvilli were key to functions such as mucus synthesis, immune system, mucosal barrier, and hormones, which breakdown and irregular brush possibly lead to fish disease (57). Thus, the weak intestinal homeostasis and lower density of microvilli in GF larvae and juveniles caused by the deficiency of microbiota, may contribute to the delayed development and inferior behavior. TEM further revealed irregular microvilli, altered mitochondria, and localized discontinuities at tight-junction regions. These three complementary modalities therefore provide convergent evidence for a microbiota-associated structural intestinal phenotype across tissue, mucus-cell, and ultrastructural scales. Thus, altered intestinal structure potentially delayed maturation, integrated with the growth, behavioral, transcriptomic, and endocrine differences as one indicator of microbiota-associated developmental phenotype. However, it is still necessary to measure permeability, transepithelial transport, or barrier failure in further in-depth functional research.

Transcriptomic profiles differed by microbial status and developmental stage, with key DEGs highlighting biological regulatory pathways that indicate a predominantly suppressive transcriptional program in the absence of microbiota. The targeted qRT-PCR connected the principal phenotypic domains of this study with focused transcriptional outcomes: HPT-axis genes with the T3/T4-related findings; GH/IGF-axis genes with growth; *PYY-a* with enteroendocrine signaling; and *cfbl*, *zgc:171509*, and *mmp13a* with immune-response features (49, 50). Increased *cdkn1a* expression in GF-7d larvae may reflect altered cell-cycle regulation, whereas differences in *guca1a* and *nr1d4b* suggest changes in signal-transduction and hormone-responsive transcription. Similarly, the pattern of differential expression suggested a dominant suppressive response in GF fish, among which the inhibition of keratin-related genes like *krt96*, ion transport (*clca1*), and transmembrane transport-related gene *slc2a6*, could reflect changes in epithelial differentiation or structural integrity, and impacts on cell functions and immune system. When developed from innate to adaptive immune, in GF-14d fish, the down-regulation of core clock genes like *per2* and *cry1b* may indicate the disruption of circadian regulation for metabolic or physiological homeostasis. Further, the 14d-specific DEGs highlighted prolonged treatment as a critical modulator, in which GF-14d vs CR-14d indicated stress-responsive pathways and growth suppression. It also implied that the ex-GF intervention may modulate key pathways differently than GF, as microbiota served as a central metabolic stressor in regulating gene expression. Notably, this stark dichotomy implied that missing microbiota profoundly redirects key physiological processes, whereas decrease of lipid metabolism genes (*lpl*, *pltp*) in GF-14d indicated adaptive metabolic shifts. In significantly changed pathways, the dominance of immune-related pathways suggested systemic activation of defense mechanisms without microbial colonization in early life of zebrafish to juveniles. The enrichment of inflammatory response, and cell and protein activity implied the disrupted immune system in GF at innate and adaptive immune stages. Additionally, GSEA captured subtle, coordinated shifts through its sensitivity to synergistic gene effects, while PPI network analysis highlighted apoptosis, inflammatory pathways, and cell cycle regulation, linking these transcriptional signatures to immune function and growth impairment.

Microbiota analysis revealed the absolute predominance of Proteobacteria phylum as well as the composition differences in early life and juvenile zebrafish; microbial rebuilding was probably driven by transplantation treatment based on GF models. The time-dependent increase of *Pseudomonas* and *Mycobacterium* aligned with known role in stress adaptation, while declined genera such as *Thermosulfidibacter* and *Acidovorax* in 14d zebrafish may indicate the niche shifts at diverse stages. The PCoA implied microbial rebuilding in early life, but the results gained from the 14d fish implied the community dynamics during maturation. The significantly changed genera in the ex-GF fish vs CR fish were increased over-time and indicated the intervention- and stage- pattern in the ex-GF models as ecological niche favoring certain microbial lineages. Thus, it highlighted that microbial transplantation can drive microbial diversification and restructuring based on the GF models and may contribute to the host development and physiological ability. Further, the enrichment of *Fluvicola* and *Deinococcus* and depletion of *Shewanella* and *Rhizobium* in ex-GF-7d fish may reflect adaptation to oxidative stress or living condition changes, while the enrichment of *Pseudomonas*, *Mycobacterium*, *Aeromonas*, and *Vibrio* in the CR-14d and ex-GF-14d group indicated their roles in environmental adaptation. These changed genera may be discovered as key biomarkers associated with microbial intervention and provided insights into host responses. The dominance of *Burkholderiales* further supported a potential role in organic matter metabolism. In the enriched pathways, the CR-7d and ex-GF-7d groups showed higher abundance of Apoptosis, Biofilm formation, and Endocytosis, while CR-14d and ex-GF-14d groups prioritized cell death mechanisms, indicating autophagy, lysosome, and Necroptosis. The activation of stem-cell signaling in CR-14d and ex-GF-14d implied novel roles in cellular regulation, highlighting the critical interplay between intervention and stages in shaping microbial functionality of zebrafish. These findings proved partial recovery is possible after recolonization and mono-association. In gnotobiotic zebrafish, colonization outcome depends on exposure timing, inoculum, persistence, community complexity, and host developmental stage (12, 53, 54); early microbial deprivation can therefore cause stage-specific host states even after later exposure. Consistent improvement of developmental, behavioral, intestinal, transcriptomic, and endocrine endpoints supported the association with microbial restoration and also indicated that a single colonization cannot substitute for the native shape of the CR microbiota.

Together with key morphological endpoints and histopathological observations, alterations in growth-promoting hormones and downstream signaling pathways further confirmed developmental delays at the regulatory level. CRH can stimulate the pituitary gland to secrete the thyroid stimulating hormone (TSH), which then stimulates the thyroid to secrete THs, i.e. the thyroid hormone (T4), and activate triiodothyronine (T3) to regulate physiological functions such as growth, development, metabolism, reproduction, and behavior (58–62). Studies on GF, SPF, and ex-GF mouse models linked microbial status to intestinal cells secretion to regulate the CRH and 5-HT concentrations, and intestinal peristalsis, thereby affecting the endocrine system and neural behavior (63–65). Additionally, it was demonstrated that PYY, CCK, ghrelin, and leptin were also important gut-brain axis hormones that involved microbial functions affecting the body’s physiological activities, weight regulation, and obesity development (66, 67). In this study, the deletion of microbiota in GF-7d and GF-14d zebrafish induced the lower content of important hormones such as GH, T3, T4, and 5-HT, and major gut-brain hormones like GLP-1, CKK, and PYY, which probably relate to the developmental delay or weakness of immune system. The GH/IGF axis regulates metabolism, somatic growth, development, reproduction, post-hatching metamorphosis, and behavior. Abnormal function of THs (T3, T4) can contribute to malformation and developmental impairments, among which the HPT axis is responsible for maintaining homeostasis of THs by regulating hormone synthesis, secretion, transport and metabolism development in embryo-larval zebrafish. The GF fish lack microbiota, which led to abnormal development of organ/tissues, such as intestinal cells and function defects, and may contribute to the inhibited hormones production and then caused growth retardation. The level of leptin showed the same trend, but ghrelin was disrupted in ex-GF-7d and GF-V-7d groups. Notably, the ex-GF and bacterial infection groups recovered to some extent but did not reach to the normal levels. The production of key metabolites such as SCFAs depends on the gut microbiota; depletion of these bacteria reduces SCFA abundance, impairing growth and behavior in zebrafish. Concomitant alterations in neuroendocrine factors, including thyroid hormone (T3) and serotonin (5-HT), further demonstrate regulation via the gut–brain axis. The gut-brain or endocrine axis regulated host health and growth and provided the novel microbial intervention strategy on zebrafish (68, 69). Finally, the expression of related genes identified the transcriptomic pattern, and the correlation analysis indicated the close inner relationships from microbiota to host health. Effects from microbial deficiency, conventional microbiota reconstruction, and bacterial colonization on intestinal epithelial-related genes showed obvious developmental stage and treatment modality dependence, with the delayed maturation of intestinal absorption- and intercellular-related phenotypes, which is consistent with observations of growth indexes and intestinal histological and ultrastructural abnormalities. Taking previous reports about gut-brain axis hormones into consideration (70), we suspected that these findings could contribute to potential biomarkers (changed bacteria, hormones, DEGs, and signalling pathways) in evaluating the health risks of shift microbiota induced by the novel pollutants’ toxicity, and microbial regulation or intervention on host growth and immune defects when developed from innate to adaptive period.

## 5 Conclusion

This study demonstrated that, compared with conventionally raised (CR) fish, germ-free (GF) zebrafish exhibit delayed growth and development across both larval and juvenile stages. The microbial rebuilding of ex-GF and bacterial infection of GF-A and GF-V fish proved that intervention could tyrestore behavioral ability, repair the damaged intestinal structure, and increase goblet cells, although these groups still did not reach the status of the unaffected groups. Moreover, transcriptomic profiling and major microbiota in zebrafish showed the potential signaling pathways and interactions between DEGs and genera. Further, the gut-brain axis hormones in GF and microbial intervention groups were altered and closely related to developmental indexes, behavioral features, histopathological score, and DEGs. Finally, these findings provided a primary microbial resource and omics background, novel insights, and important protocols for the generation and application of GF animal models and microbial strategies in evaluating the impacts of emerging pollutants on intestinal homeostasis, as well as the probiotics screen from gut bacterial strains, and biomarker research regions. However, conventional community recolonization and mono-association produced partial, endpoint-, and stage-dependent recovery, with a gap remaining between the complete microbial and developmental degree as CR fish. Thus, more investigations into intestinal functions, immune response, and the mechanism of regulatory pathways are still needed.

## Data Availability

The datasets presented in this study can be found in online repositories. The names of the repository/repositories and accession number(s) can be found in the article/**Supplementary Material**.
