# Supplementary material for: Microbial intervention on germ-free zebrafish (Danio rerio) developmental delay and gut-brain axis hormones disturbance from innate to adaptive immune stages
Source: Front Immunol. 2026 Oct 19;17:1930616. doi: 10.3389/fimmu.2026.1930616 (PMC13724341; doi:10.3389/fimmu.2026.1930616)
Supplement: Supplementary file 1 [file DataSheet1.docx]

**Supplementary Information**

**Microbial intervention on germ-free zebrafish (*Danio rerio*) developmental delay and gut-brain axis hormones disturbance from innate to adaptive immune stages**

Lan-Chen Zhang^1^^†^, Hao-Yu Yang^1†^, Jin-Qiu Jia^1†^, Xiao-Bing Li^1,2†^, Jia-Xin He^1^, Ying-Tang^1^, Yi-Wei Tang^1^, Pan-Pan Jia^1^*

^1^College of Public Health, Chongqing Medical University, Chongqing 400016, China

^2^Science and Technology Industry Development Center, Chongqing Medical and Pharmaceutical College, Chongqing 401331, China

^†^The authors contributed equally to this work.

^*^Corresponding author.

E-mails: jiapanpan@cqmu.edu.cn (P.P.J), and jia_panpan@126.com

**Text S1. Chemicals, materials, and preparation.** NaCl (CAS 7647-14-5), soy peptone (Coolaber, Cat. CS10383), tryptone (Oxoid, Cat. LP0042), and agar powder (Solarbio, CAS 9002-18-0) were used to prepare tryptic soy agar (TSA) plates and tryptic soy broth (TSB). Sterile defibrinated sheep blood (Solarbio, Cat. TX0030) was added to TSA to prepare 5% blood agar plates. Brain heart infusion medium (BHI, Solarbio, Cat. B8130), glycerol (>99.0%; CAS 56-81-5; Cat. G8190), and a bacterial genomic DNA extraction kit (Solarbio, Cat. D1600) were also used. Taq polymerase reagents (Takara, Cat. R001A) and the bacterial genomic DNA extraction kit were used for conventional PCR assays of bacterial isolates and GF-model samples. MS-222 (3-aminobenzoic acid ethyl ester methanesulfonate; 98%) was obtained from Aladdin. All other reagents were of analytical grade.

**Text S2. Generation, maintenance, and validation of germ-free (GF) zebrafish.** Embryos were washed, and debris and dead or unfertilized eggs were removed. At 2 hpf, embryos were transferred to sterilized plates containing AB-GZM and cultured at 28.5 °C for 6–8 h. Embryos were rinsed three times with AB-GZM within 10 min, immersed in 0.2 g/L povidone-iodine for 1 min, rinsed three times, treated with 0.02 g/L sodium hypochlorite for 15 min under gentle agitation, and rinsed three additional times, following our previous protocol. Embryos were maintained in sterile six-well plates at 28 ± 0.5 °C under a 14 h light/10 h dark photoperiod. Each treatment group was established across two to three six-well plates with approximately eight embryos or fish per well. Medium was renewed daily. From 8 dpf, GF fish were fed sterile egg yolk mixed with GF *Artemia* sp. once daily. Survival, hatching, heart rate, body length, and body weight were recorded. Every well was screened daily for contamination; contaminated wells were excluded from the GF cohort and were not used for GF sampling or analysis.

Sterility was assessed daily for every well and again at experimental sampling. For culture-based testing, 20-50 μL of spent medium was spread onto TSA and 5% blood TSA plates under aerobic and anaerobic conditions, and 200 μL was inoculated into aerobic and anaerobic BHI and TSB tubes. Cultures were maintained at 30 °C; aerobic broths were agitated at 150–180 rpm. Plates and broths were examined from 24 h through 7 days, and a negative result required no colonies on solid medium and no turbidity in broth. Spent medium was also examined by bacterial 16S rRNA PCR using primers 27F and 1492R. Each 20-μL reaction contained 1 μL template, 1 μL of each 10 μM primer, and a commercial Taq polymerase master mixture. Cycling comprised 95 °C for 4 min; 35 cycles of 94 °C for 1 min, 55 °C for 1 min, and 72 °C for 1 min; and 72 °C for 10 min. Products were examined on 1% agarose gels for the expected 1,465-bp band. DNA from CR culture water and nuclease-free water served as positive and negative controls, respectively. A batch was classified as germ free only when all culture and PCR assays were negative. Any contaminated well was excluded immediately and avoid to be used as GF sample.

**Text S3. Preparation of samples for transmission electron microscopy (TEM).** Six to eight fish per group were sampled for ultrastructural processing with prefixed in 2.5% glutaraldehyde at 4 °C, rinsed in phosphate buffer, and postfixed in 1% OsO₄ for 1 h at 4 °C. Samples were dehydrated through a graded ethanol series, infiltrated, and embedded in epoxy resin. Ultrathin sections (60–80 nm) were collected on copper grids and contrasted with uranyl acetate and lead citrate before TEM examination. Images were acquired at 2.0k× and 7.0k× from comparable intestinal regions. Thirty sections per group were examined.

**Text S4. Measurement of behavioral ability in CR, GF, ex-GF, and mono-associated fish.** Six fish per group were recorded at 7 and 14 dpf using a DanioVision system and EthoVision XT software. Fish were placed individually in 24-well plates containing sterile water at 28 ± 0.5 °C. Outcomes included distance traveled, velocity, acceleration, movement state, and activity. Activity was classified as manic (>60%), active (20–60%), or static (<20%). Fish acclimated for 10 min in darkness before testing. The light–dark test comprised five consecutive 5-min periods: dark, light, dark, light, and dark. Trajectory and heat-maps show representative recordings, whereas the summary plots report the six analyzed fish per group.

**Text S5. Library preparation and quality control for transcriptome sequencing.** Total RNA was extracted from three independently biological pools per group, with 30 fish per pooled sample. Sequencing libraries were generated using NEBNext® UltraTM RNA Library Prep Kit for Illumina® (NEB, USA) following manufacturer’s recommendations and index codes were added to attribute sequences to each sample. Briefly, mRNA was purified from total RNA using poly-T oligo-attached magnetic beads. Fragmentation was carried out using divalent cations under elevated temperature in NEBNext First Strand Synthesis Reaction Buffer(5X). First strand cDNA was synthesized using random hexamer primer and M-MuLV Reverse Transcriptase. Second strand cDNA synthesis was subsequently performed using DNA Polymerase I and RNase H. Remaining overhangs were converted into blunt ends *via* exonuclease/polymerase activities. In order to select cDNA fragments of preferentially 250~300 bp in length, the library fragments were purified with AMPure XP system (Beckman Coulter, Beverly, USA). Then 3 µL USER Enzyme (NEB, USA) was used with size-selected, adaptor-ligated cDNA at 37°C for 15 min followed by 5 min at 95°C before PCR was performed with Phusion High-Fidelity DNA polymerase, Universal PCR primers and Index (X) Primer. At last, PCR products were purified (AMPure XP system) and library quality was assessed on the Agilent Bioanalyzer 2100 system. The clustering of the index-coded samples was performed on a cBot Cluster Generation System using TruSeq PE Cluster Kit v3-cBot-HS (Illumia) according to the manufacturer’s instructions. After cluster generation, the library preparations were sequenced on an Illumina platform and 150 bp paired-end reads were generated.

**Text S6. Transcriptome processing, differential-expression genes, and enrichment analysis.** Adapter-containing reads, reads with excessive ambiguous bases, and low-quality reads were removed before alignment. Paired-end clean reads were aligned to the *Danio rerio* reference genome and corresponding gene annotation used for the deposited analysis, and gene-level raw integer counts were generated. Differential expression was analyzed from the raw integer count matrix using DESeq2 with treatment group as the design factor. *P* values were adjusted using the Benjamini-Hochberg method, and genes with adjusted *P* < 0.05 and |log2 fold change| >= 2 were considered differentially expressed. Genes with negligible counts across all samples were excluded before model fitting. GO and KEGG enrichment used the detected-gene set as background were corrected using the Benjamini-Hochberg method to understand high-level functions and utilities of the biological system.

**Text S7.** **16S rRNA sequencing and analysis of microbiota in zebrafish.** Total DNA was extracted from whole-body zebrafish samples, and the V3–V4 region was amplified with primers 341F (5′-CCTAYGGGRBGCASCAG-3′) and 806R (5′-GGACTACNNGGGTATCTAAT-3′). PCR consisted of 98 °C for 1 min; 30 cycles of 98 °C for 10 s, 50 °C for 30 s, and 72 °C for 30 s; and 72 °C for 5 min. Purified amplicons were used to prepare indexed libraries with the NEBNext Ultra DNA Library Prep Kit and were sequenced on an Illumina NovaSeq instrument as 250-bp paired-end reads. Barcodes and primers were removed with cutadapt v3.3 (--minimum-length 20), paired reads were merged with FLASH v1.2.11 (--min-overlap 10; --max-mismatch-density 0.2), and tags were filtered with fastp v0.23.1 (-q 19; -u 15). Chimeras were removed with VSEARCH v2.16.0. ASVs were inferred with the DADA2 plugin in QIIME 2 v2022.2 and annotated against the SILVA reference database. Relative abundances were used for taxonomic summaries, and diversity analyses used a depth-normalized ASV table. Bray–Curtis PCoA was generated in R v4.0.3 and interpreted descriptively. Differential abundance was assessed with DESeq2 using Benjamini–Hochberg-adjusted *P* values; LEfSe v1.1.01 used an LDA threshold >2.0. PICRUSt2 v2.3.0 was used to predict functional potential. Functional comparisons used one-way ANOVA followed by Duncan’s test.

**Text S8.** **Measurement of whole-body hormones and short-chain fatty acids by ELISA kits.** Samples were weighed and homogenized in PBS (1x, pH 7.4) at approximately 9 mL/g with protease inhibitor. Homogenates were centrifuged at 5,000 x g for 5 min, and the supernatants were stored at -80 ^o^C. GH, T3, T4, 5-HT, GLP-1, CCK, PYY, ghrelin, and leptin were quantified using the commercial kits, following the supplied assay protocols. Absorbance was read at 450 nm after incubation, and concentrations were calculated from assay-specific standard curves. Total SCFAs were measured from clarified whole-body supernatants using the corresponding absorbance-based total-SCFA assay and supplied standard. Three replicate biological samples per group were detected twice (n = 6 values per group). Hormones were detected with ng/mL or pg/mL levels, and total SCFAs at nmol/mL. All groups were processed with the same homogenization, assay procedure, plate reading, and assay-specific standard-curve calculation, so the comparison supported the relative whole-body assay readout among experimental groups.

**Table S1. Expression level of key gene markers searched from zebrafish transcriptomic profile**

| **Features** | **baseMean** | **log2FoldChange** | **lfcSE** | **stat** | **pvalue** | **padj** | **Gene Symbol** | **description** | **STRING Protein description** |
| --- | --- | --- | --- | --- | --- | --- | --- | --- | --- |
| GF_7d.vs.CR_7d | | | | | | | | | |
| ENSDARG00000054155 | 917.0646 | -0.41297 | 0.225701 | -1.82974 | 0.067289 | 0.711613 | pcna | proliferating cell nuclear antigen | Proliferating cell nuclear antigen; This protein is an auxiliary protein of DNA polymerase delta and is involved in the control of eukaryotic DNA replication by increasing the polymerase's processibility during elongation of the leading strand; Belongs to the PCNA family |
| ENSDARG00000091150 | 372.9387 | -0.01012 | 0.254134 | -0.03982 | 0.96824 | 0.999901 | mki67 | marker of proliferation Ki-67 | Marker of proliferation Ki-67 |
| ENSDARG00000114554 | 109.3803 | -1.0465 | 0.628862 | -1.66411 | 0.09609 | 0.801522 | cdx1a | caudal type homeobox 1a | --- |
| ENSDARG00000116139 | 255.2364 | -0.77665 | 0.373796 | -2.07774 | 0.037733 | 0.56154 | cdx1b | caudal type homeobox 1 b | --- |
| ENSDARG00000040466 | 1678.107 | -0.25837 | 0.283707 | -0.9107 | 0.362452 | 0.999901 | vil1 | villin 1 | Villin 1 |
| ENSDARG00000006427 | 2136.09 | 0.677909 | 0.401118 | 1.690048 | 0.091019 | 0.78617 | fabp2 | fatty acid binding protein 2, intestinal | Fatty acid binding protein 2, intestinal; Belongs to the calycin superfamily. Fatty-acid binding protein (FABP) family |
| ENSDARG00000005108 | 765.0735 | -0.62039 | 0.296669 | -2.09119 | 0.036511 | 0.555248 | oclna | occludin a | May play a role in the formation and regulation of the tight junction (TJ) paracellular permeability barrier. |
| ENSDARG00000003091 | 451.137 | -0.6719 | 0.304265 | -2.20826 | 0.027226 | 0.495272 | oclnb | occludin b | Occludin b |
| ENSDARG00000077506 | 4099.603 | -0.15394 | 0.265606 | -0.5796 | 0.562186 | 0.999901 | tjp1a | tight junction protein 1a | Tight junction protein 1a; Belongs to the MAGUK family |
| ENSDARG00000042357 | 329.0156 | -0.5604 | 0.170463 | -3.28751 | 0.001011 | 0.070355 | cldnk | claudin k | Claudin; Plays a major role in tight junction-specific obliteration of the intercellular space, through calcium- independent cell-adhesion activity |
| ENSDARG00000030723 | 114.4176 | -0.52834 | 0.228474 | -2.31245 | 0.020753 | 0.43586 | cldn11b | claudin 11b | Claudin; Plays a major role in tight junction-specific obliteration of the intercellular space, through calcium- independent cell-adhesion activity |
| ENSDARG00000016081 | 394.7486 | -0.39056 | 0.415325 | -0.94036 | 0.347031 | 0.999901 | cldn15la | claudin 15-like a | Claudin; Plays a major role in tight junction-specific obliteration of the intercellular space, through calcium- independent cell-adhesion activity |
| GF_14d.vs.CR_14d | | | | | | | | | |
| ENSDARG00000054155 | 917.0646 | 0.97171 | 0.223128 | 4.354952 | 1.33E-05 | 0.000374 | pcna | proliferating cell nuclear antigen | Proliferating cell nuclear antigen; This protein is an auxiliary protein of DNA polymerase delta and is involved in the control of eukaryotic DNA replication by increasing the polymerase's processibility during elongation of the leading strand; Belongs to the PCNA family |
| ENSDARG00000091150 | 372.9387 | 2.184481 | 0.258699 | 8.44411 | 3.06E-17 | 1.59E-14 | mki67 | marker of proliferation Ki-67 | Marker of proliferation Ki-67 |
| ENSDARG00000114554 | 109.3803 | -1.90431 | 0.655907 | -2.90332 | 0.003692 | 0.032982 | cdx1a | caudal type homeobox 1a | --- |
| ENSDARG00000116139 | 255.2364 | -2.22349 | 0.437017 | -5.08787 | 3.62E-07 | 1.76E-05 | cdx1b | caudal type homeobox 1 b | --- |
| ENSDARG00000040466 | 1678.107 | -0.33229 | 0.290959 | -1.14206 | 0.253431 | 0.53282 | vil1 | villin 1 | Villin 1 |
| ENSDARG00000006427 | 2136.09 | 0.35709 | 0.40265 | 0.886849 | 0.37516 | 0.653237 | fabp2 | fatty acid binding protein 2, intestinal | Fatty acid binding protein 2, intestinal; Belongs to the calycin superfamily. Fatty-acid binding protein (FABP) family |
| ENSDARG00000005108 | 765.0735 | -1.60675 | 0.300707 | -5.34324 | 9.13E-08 | 5.41E-06 | oclna | occludin a | May play a role in the formation and regulation of the tight junction (TJ) paracellular permeability barrier. |
| ENSDARG00000003091 | 451.137 | -2.66925 | 0.315295 | -8.46586 | 2.54E-17 | 1.37E-14 | oclnb | occludin b | Occludin b |
| ENSDARG00000077506 | 4099.603 | -0.12359 | 0.265775 | -0.46502 | 0.64192 | 0.835414 | tjp1a | tight junction protein 1a | Tight junction protein 1a; Belongs to the MAGUK family |
| ENSDARG00000036376 | 239.8853 | -2.07488 | 0.296975 | -6.98672 | 2.81E-12 | 6.12E-10 | cldn7a | claudin 7a | Claudin-7-A; Plays a major role in tight junction-specific obliteration of the intercellular space |
| ENSDARG00000043128 | 1857.808 | -2.42157 | 0.351247 | -6.89421 | 5.42E-12 | 1.13E-09 | cldne | claudin e | Claudin; Plays a major role in tight junction-specific obliteration of the intercellular space, through calcium- independent cell-adhesion activity |
| ENSDARG00000016081 | 394.7486 | -0.90869 | 0.42667 | -2.12972 | 0.033195 | 0.154821 | cldn15la | claudin 15-like a | Claudin; Plays a major role in tight junction-specific obliteration of the intercellular space, through calcium- independent cell-adhesion activity |

Note: 1.The gene expression of Top several mutants of *olcn*, *cldn* family were listed. 2. Yellow marker means that the gene was in DEGs set.

**Figure S1.** **The identification of germ-free zebrafish models by using various methods.** (A-B) Detection of daily samples on the aerobic solid medium TSA plates. (C) Sterility testing results of the aerobic liquid medium TSB tubes. (D) Sterility testing results of the anaerobic liquid medium TSB tubes.

**Figure S2. The GSEA of germ-free zebrafish models compared with CR fish.** (A-B) Significant changed signaling pathways in GF fish at larvae and juvenile stages, which highlighted the microbial intervention on the immune response, cell migration, peptidase activity, and GTP binding.

**Figure S3. Validation of key genes’ expression related to cell proliferation, epithelial differentiation, and intestinal barrier maturation in zebrafish.** Relative expression level of critical genes *pcna*, *mki67*, *cdx1b*, *fabp2*, *oclna*, and *cldn15la* in 7d (A, B) groups, showing the inhibition trend by GF treatment. The expression of *pcna*, *mki67*, *fabp2* showed the increase level in 14d (C, D) groups, but *oclna* and *cldn15la* were still inhibited. The *, **, *** of *P*<0.05, 0.01, 0.001 indicated significant differences between treated groups and corresponding control groups.
